# Glycoprotein (GP)96 Is Essential for Maintaining Intestinal Epithelial Architecture by Supporting Its Self-Renewal Capacity

**DOI:** 10.1016/j.jcmgh.2022.12.004

**Published:** 2022-12-11

**Authors:** Janine Häfliger, Marlene Schwarzfischer, Kirstin Atrott, Claudia Stanzel, Yasser Morsy, Marcin Wawrzyniak, Silvia Lang, Tomas Valenta, Konrad Basler, Gerhard Rogler, Michael Scharl, Marianne R. Spalinger

**Affiliations:** 1 Department of Gastroenterology and Hepatology, University Hospital Zurich, University of Zurich, Zurich, Switzerland; 2 Department of Molecular Life Sciences, University of Zurich, Zurich, Switzerland

**Keywords:** Intestinal Stem Cells, Wnt Signaling, Notch Signaling, ER Stress, LRP6, BIP, Immunoglobulin Binding Protein, BSA, bovine serum albumin, CHIR99021, glykogen synthase kinase 3 inhibitor, DAPT, N-[N-(3, 5-difluorophenacetyl)-l-alanyl]-s-phenylglycinet-butyl ester, ER, endoplasmic reticulum, GP, glycoprotein, GRP, glucose-regulated protein, HBSS, Hank’s balanced salt solution, IBD, inflammatory bowel disease, IEC, intestinal epithelial cell, ISC, intestinal stem cell, KO, knockout, LRP, low-density lipoprotein-receptor–related protein, LYZ, lysozyme, OLFM4, Olfactomedin 4, PBS, phosphate-buffered saline, PCR, polymerase chain reaction, TA, transit-amplifying, UPR, unfolded protein response, VPA, valproic acid, Wnt, Wingless/Integrated, WT, wild-type, XAV, tankyrase1/2 inhibitor, 4-OHT, (Z)-4-hydroxytamoxifen

## Abstract

**Background & Aims:**

Glycoprotein (GP)96 is an endoplasmic reticulum–resident master chaperone for cell surface receptors including the Wnt co-receptors low-density lipoprotein-receptor–related protein 5/6. Intestinal epithelial cell (IEC)-specific deletion of Gp96 is embryonically lethal. However, the role of GP96 in adult intestinal tissue and especially within the intestinal stem cell (ISC) niche is unknown. Here, we investigated how GP96 loss interferes with intestinal homeostasis by compromising viability, proliferation, and differentiation of IECs.

**Methods:**

Tamoxifen was used to induce Cre-mediated deletion of *Gp96* in GP96-Villin^creERT2^ (Cre recombinase-Estrogen-Receptor Transgene 2) mice and intestinal organoids. With H&E and immunofluorescence staining we assessed alterations in intestinal morphology and the presence and localization of IEC types. Real-time polymerase chain reaction and Western blot analysis were performed to explore the molecular mechanisms underlying the severe phenotype of *Gp96* KO mice and organoids.

**Results:**

IEC-specific deletion of *Gp96* in adult mice resulted in a rapid degeneration of the stem cell niche, followed by complete eradication of the epithelial layer and death within a few days. These effects were owing to severe defects in ISC renewal and premature ISC differentiation, which resulted from defective Wnt and Notch signaling. Furthermore, depletion of GP96 led to massive induction of endoplasmic reticulum stress. Although effects on ISC renewal and adequate differentiation were partly reversed upon activation of Wnt/Notch signaling, viability could not be restored, indicating that reduced viability was mediated by other mechanisms.

**Conclusions:**

Our work shows that GP96 plays a fundamental role in regulating ISC fate and epithelial regeneration and therefore is indispensable for maintaining intestinal epithelial homeostasis.

**Summary**

We report the fundamental requirement of glycoprotein 96 for intestinal self-renewal and regeneration. Deletion of *Gp96* in intestinal epithelial cells impairs Wnt/Notch signaling and endoplasmic reticulum homeostasis, resulting in degeneration of the stem cell niche, and, eventually, loss of epithelial integrity.

The intestinal epithelium is characterized by a remarkable self-renewal capacity and a continuously high turnover of intestinal epithelial cells (IECs), which originate from intestinal stem cells (ISCs).^1^^,^^2^ A single layer of highly specialized IECs form a tight but selective barrier that allows nutrient uptake, while preventing potentially harmful agents such as bacteria and food antigens from invading the host.^3^ Defects in proliferation and differentiation of ISCs into mature IECs result in impaired barrier function, which is a hallmark of several disorders of the gastrointestinal tract, including inflammatory bowel disease (IBD).^4^^,^^5^

Glycoprotein (GP)96, also referred to as *GPR94* and *HSP90B1*, is an endoplasmic reticulum (ER) resident protein that acts as a master chaperone for cell surface receptors, including Toll-like receptors, integrins, and the Wingless/Integrated (Wnt) co-receptor low-density lipoprotein receptor-related protein (LRP)6.^6^, ^7^, ^8^ Full-body and IEC-specific deletion of *Gp96* results in developmental defects and embryonic lethality.^9^^,^^10^ However, the physiologic role of GP96 in adult intestinal tissue and especially its function for the renewal capacity of the epithelium is not yet fully understood. It has been shown that in the absence of GP96, the Wnt co-receptor LRP6 is not properly exported from the ER to the cell surface, resulting in defective cell membrane localization.^9^ Canonical Wnt signaling through the Frizzled-LRP5/6 receptor complex is crucial for ISC control and maintenance.^11^^,^^12^ Wnt ligands secreted by adjacent Paneth cells and other cells surrounding ISCs^13^, ^14^, ^15^, ^16^, ^17^ bind to the Frizzled-LRP5/6 receptor complex, which ultimately leads to the accumulation and nuclear translocation of β-catenin that promotes ISC survival and maintenance of stemness.^11^^,^^18^^,^^19^ Mice lacking the Wnt ligand co-receptors LRP5/6 in IECs die within 1 day after birth, presumably because of defective Wnt target gene expression, proliferation inhibition, and premature differentiation of crypt stem cells, leading to progressive loss of IECs.^20^ Interestingly, tamoxifen-induced full-body deletion of *Gp96* in Hsp90b1^flox/flox^;Rosa26-Cre-recombinase Estrogen receptor trangene 2(^creERT^^)^ mice resulted in a similar intestinal phenotype, including reduced β-catenin accumulation and defective proliferation of IECs.^9^

In addition to Wnt/β-catenin signaling, multiple additional cellular pathways including Notch, ER stress, epidermal growth factor receptor, Eph/ephrin, Hippo, and bone morphogenetic protein are involved in coordinating the dynamic balance between proliferation, differentiation, and apoptosis of IECs.^21^ Active Notch signaling is essential for ISC renewal and regulates the fate of transit-amplifying (TA) cells by promoting the generation of absorptive enterocytes.^22^ Compromised Notch signaling leads to an overall increase in secretory cell types and depletion of proliferative stem and progenitor cells. Notch-receptor signaling activates the transcription of targets genes such as *Hes1*. *Hes1* expression represses the transcriptional activity of *Atoh1*, thereby restricting secretory cell commitment.^23^ Thus, the homeostatic pattern of absorptive and secretory cells in the intestinal epithelium largely depends on functional Notch signaling.^24^

Although previous studies have focused primarily on the effect of Wnt and Notch signaling individually, it is becoming increasingly evident that the synergistic and antagonistic interplay of these pathways regulates ISC maintenance and cell fate. For instance, it has been proposed that one of the main functions of Notch signaling in ISC maintenance is attenuating Wnt signaling to a level that supports stem cell activity and prevents secretory cell metaplasia.^25^ Mechanistically, this antagonistic modulation of Wnt activity depends on the interaction of Notch with the active form of β-catenin.^26^ By negatively regulating β-catenin activity and titrating the Wnt/β-catenin signaling output, Notch signaling tempers ISC proliferation. Furthermore, active Wnt signaling in ISCs suppress mitogen-activated protein kinase signaling and thereby prevents premature differentiation into TA cells.^27^ Thus, IEC fate decision depends on a complex integration of multiple signaling and spatial cues that ultimately ensure a balanced and homeostatic turnover of the intestinal epithelium.

In addition to its effect on Wnt signaling, depletion of the molecular chaperone GP96 is known to promote ER stress.^10^ In homeostatic conditions, ER stress is present in ISCs only at low levels, and the increased sensitivity to ER stress in these cells induces transition to TA cells and loss of self-renewal capacity.^28^ This indicates that GP96 might interfere with several events that determine ISC fate and the renewal capacity of the intestinal epithelium. In addition to its reported role on Wnt signaling, however, the precise mechanism by which GP96 interferes with intestinal homeostasis and ISC fate decision is not known.

In this study, we investigated the role of GP96 in the intestinal epithelium by characterizing the time-dependent effects on epithelial morphology, cell viability, proliferation, and differentiation upon IEC-specific GP96 depletion in adult mice. We show that loss of GP96 results in degeneration of the stem cell niche, followed by complete eradication of the epithelial layer. In mice, IEC-specific deletion of *Gp96* and subsequent disruption of the epithelial layer results in death within a few days. We show that GP96 depletion not only affects Wnt signaling, but also impairs Notch signaling, which, in combination with defective handling of ER stress, resulted in severe defects in epithelial regeneration.

## Results

### IEC-Specific GP96 Depletion Severely Impairs the Integrity of the Small Intestinal Epithelium and Leads to a Lethal Phenotype in Mice

**Figure 1:**
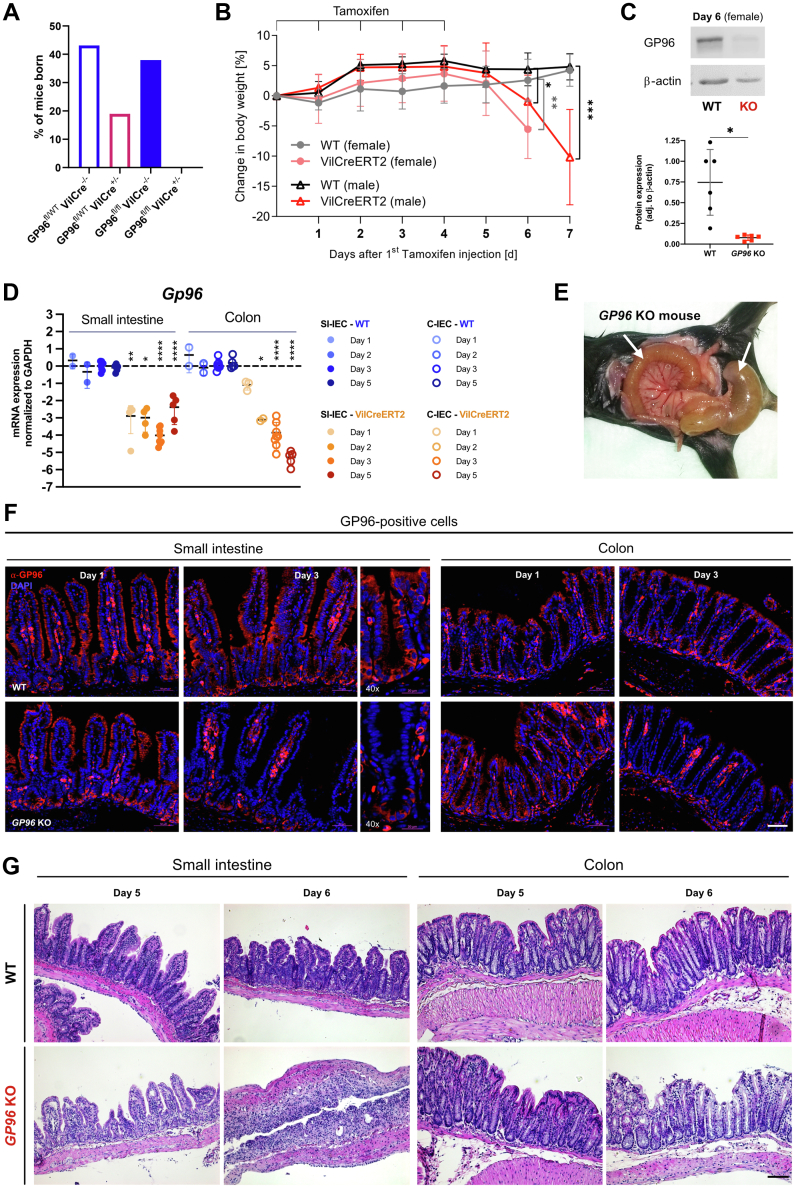
**IEC-specific GP96 depletion results in rapid weight loss within 6 days after the first tamoxifen injection and complete eradication of the small intestinal epithelium.** (*A*) Percentage of viable pups born with the indicated genotypes. (*B*) GP96^fl/fl^ mice (WT) and GP96^fl/fl^-Villin^creERT2^ littermates (*Gp96* KO) were injected intraperitoneally with tamoxifen for 5 consecutive days and killed 1–7 days after the first tamoxifen injection. Experimental timeline and weight development after first tamoxifen injection. N = 3–8 mice/group. (*C*) Representative Western blot for GP96 of small intestinal epithelial cells. (*D*) Relative messenger RNA (mRNA) expression in small (SI-IEC) and large (C-IEC) intestinal epithelial cells, normalized to glyceraldehyde-3-phosphate dehydrogenase (GAPDH) and treated WT control mice on days 1, 2, 3, and 5. N = 2–7 mice/group. (*E*) Representative image of a transparent and fragile-appearing intestine in GP96-Villin^creERT2^ (*Gp96* KO) mice. (*F*) Small intestinal and colonic tissue sections from days 1 to 3 stained for GP96 (red) and counterstained with 4′,6-diamidino-2-phenylindole (DAPI) (blue). *Scale bars*: 50 μm in pannels with 10x maginifications or 20 μm in the panels with higher magnification (×40) of the indicated sections. (*G*) Representative pictures from H&E-stained ileum (*left*) and colon (*right*) sections from days 5 and 6. *Scale bar*: 100 μm. Significance was calculated using multiple *t* tests (reverse-transcription PCR) or unpaired *t* test (body weight change). *Bars* represent means with SD. *Asterisks* indicate significant differences, as follows: ∗*P* ≤ .05, ∗∗*P* ≤ .01, ∗∗∗*P* ≤ .001, and ∗∗∗∗*P* ≤ .0001. SI, small intestinal.

In line with previous reports, mice with homozygous knockout (KO) of *Gp96* in IECs were not viable (Figure 1*A*). In addition, mice that expressed only 1 allele of GP96 in IECs (GP96^fl/-^-Cre^+/-^ mice) were born at a lower frequency when compared with controls (Figure 1*A*), indicating a critical role for GP96 in IECs. To study the role of GP96 in the intestinal epithelium, we generated GP96^fl/fl^-Villin^creERT2^ mice, which allow for a timed, tamoxifen-induced *Gp96* deletion in IECs. Littermates with a loxP flanked *Gp96* gene without the Villin^creERT2^ construct served as controls and the efficiency of the *Gp96* KO was verified by gene expression analysis, Western blot on intestinal IECs, and immunofluorescence staining for GP96 (Figure 1*C*, *D*, and *F*).

**Figure 2:**
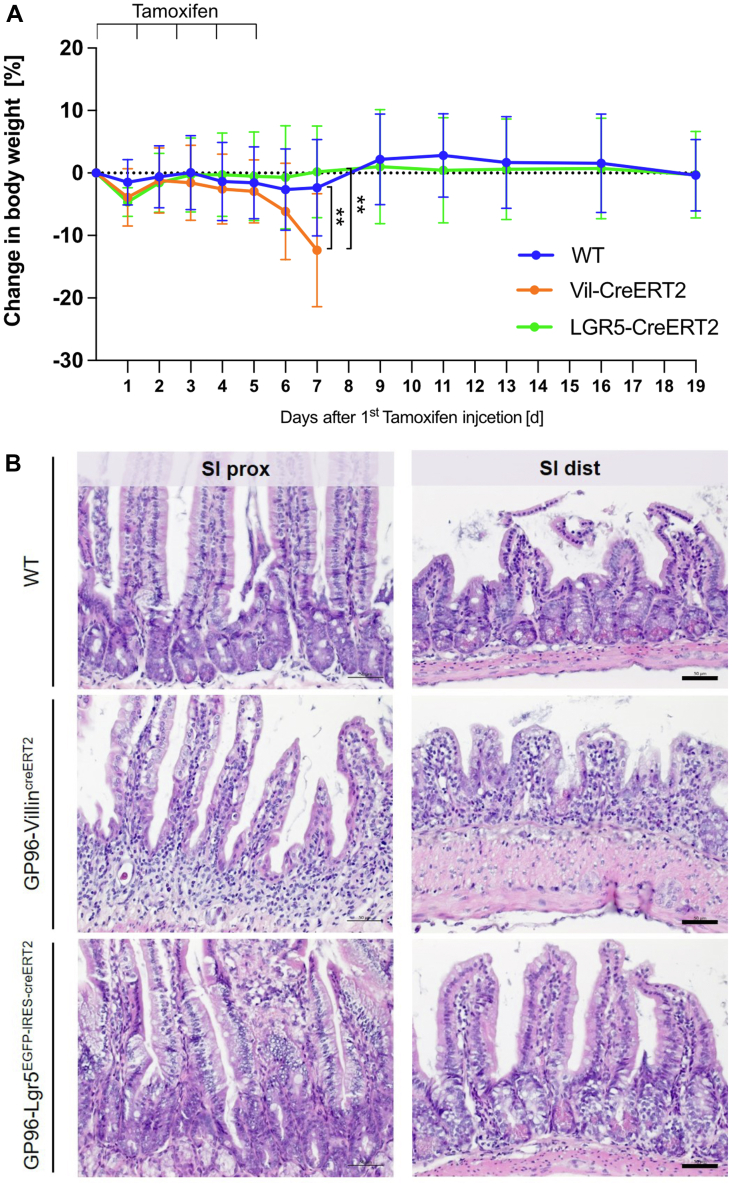
**GP96-Lgr5**^**EGFP-IRES-creERT2**^**mice did not show a phenotype after tamoxifen injection.** (*A*) GP96^fl/fl^ mice (WT), GP96^fl/fl^-Villin^creERT2^ littermates (Vil-CreERT2), and GP96-Lgr5^EGFP-IRES-creERT2^ (LGR5-CreERT2) were injected intraperitoneally with tamoxifen for 5 consecutive days and killed 1–19 days after the first tamoxifen injection. Experimental timeline and weight development after first tamoxifen injection. N = 4–6 mice/group. (*B*) Representative pictures of H&E-stained proximal (SI prox) and distal (SI dist) small intestinal sections from day 7. *Scale bars*: 50 μm. Significance was calculated with an unpaired *t* test. *Bars* represent mean with SD. *Asterisks* indicate significant differences, as follows ∗*P* ≤ .05, ∗∗*P* ≤ .01, ∗∗∗*P* ≤ .001, and ∗∗∗∗*P* ≤ .0001.

**Figure 3:**
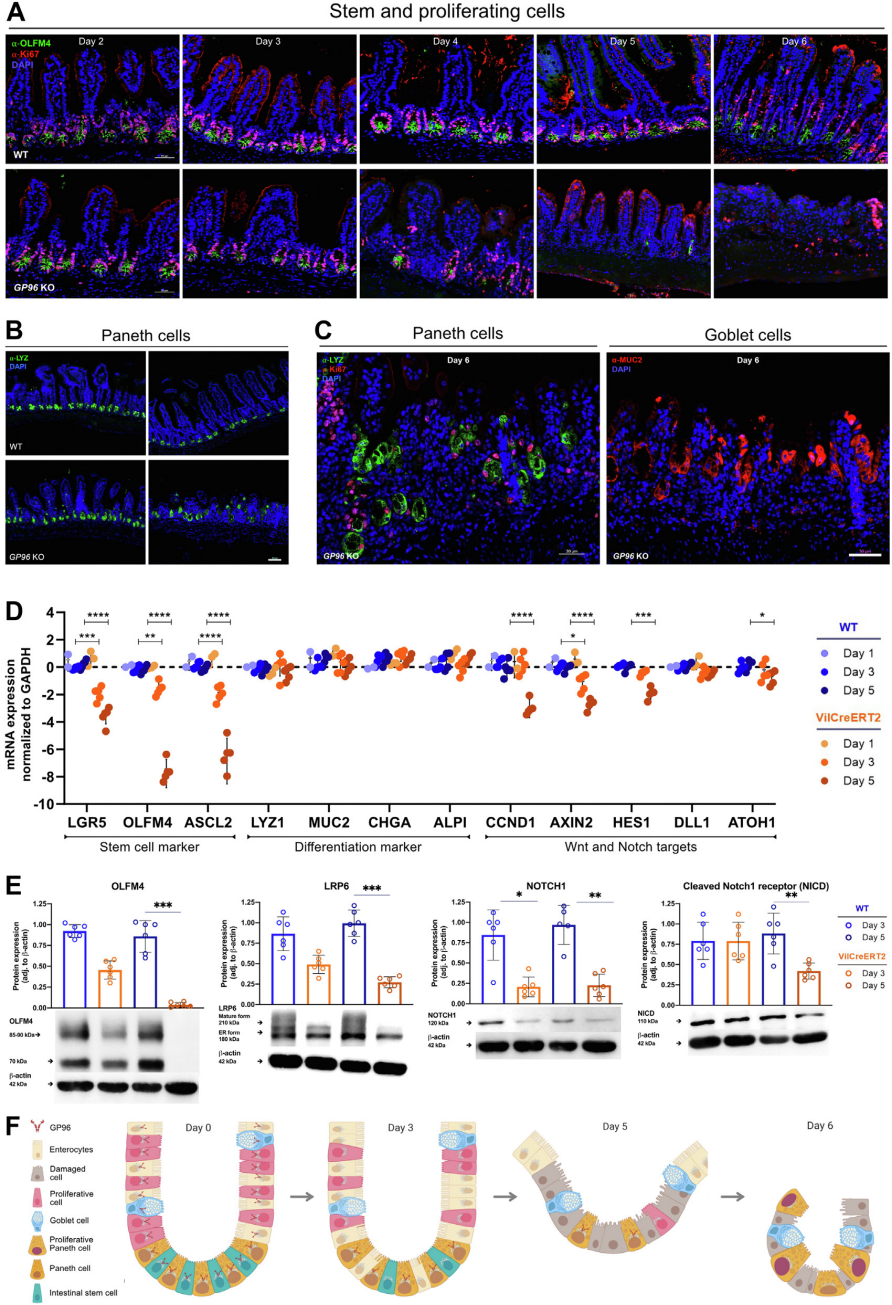
**IEC-specific *Gp96* deletion leads to a continuous loss of proliferating cells, ISCs, and Paneth cells, and alters the morphology and location of secretory cell types.** Small intestinal tissue sections of GP96^fl/fl^ (WT) and GP96^fl/fl^-Villin^creERT2^ (*Gp96* KO) mice were collected on days 2–6 after first tamoxifen-injection and stained for (*A*) OLFM4 (green) and Ki67 (red); (*B*) lysozyme (green); or (*C*) lysozyme (green), Ki67 (red), and MUC2 (red) in combination. *Scale bars*: 20 μm (*B*), 50 μm (*A* and *C*). (*D*) Quantitative reverse-transcription PCR analysis in small intestinal epithelial cells, normalized to glyceraldehyde-3-phosphate dehydrogenase (GAPDH) and WT control mice on days 1, 3, and 5. N = 2–7 mice/group. (*E*) Western blot analysis on lysates from small intestinal epithelial cells collected on days 3 and 5 after first tamoxifen injection, normalized to β-actin. Graphs show representative Western blot images from each experimental group. N = 6 mice/group. (*F*) Schematic representation of the main findings of this figure showing the loss of the stem cell niche and the appearance of morphologically altered and mislocated Paneth and goblet cells as a consequence of GP96 depletion. Created by BioRender. Significance was calculated using 2-way analysis of variance with a Tukey correction (reverse-transcription PCR) or a Kruskal–Wallis test (1-way analysis of variance, nonparametric) (Western blot). *Bars* represent mean with SD. *Asterisks* indicate significant differences, as follows: ∗*P* ≤ .05, ∗∗*P* ≤ .01, ∗∗∗*P* ≤ .001, and ∗∗∗∗*P* ≤ .0001. DAPI, 4′,6-diamidino-2-phenylindole; mRNA, messenger RNA; NICD, Notch intracellular domain.

**Figure 4:**
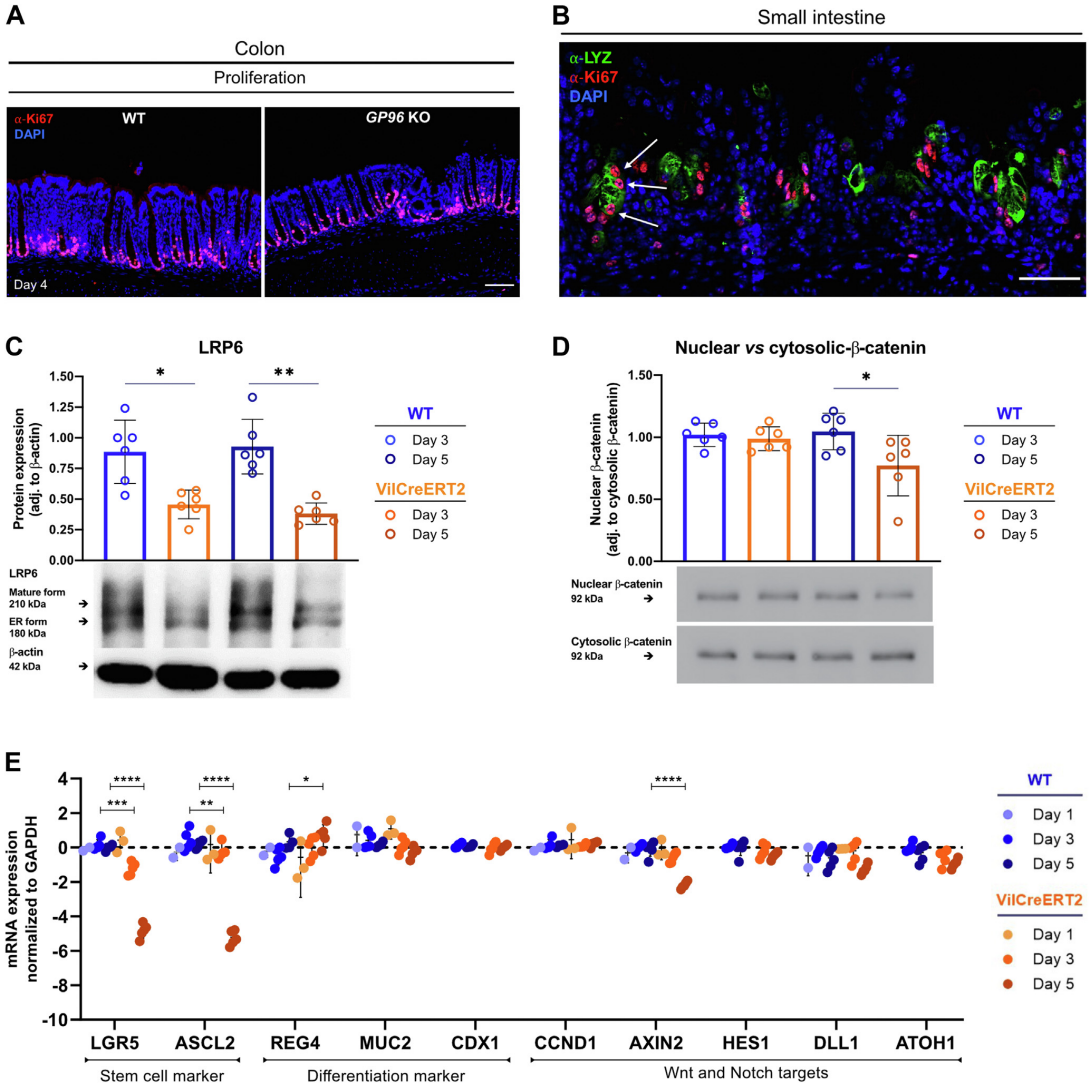
**Compared with the small intestine, depletion of GP96 in colonic IECs leads to a similar but less-pronounced in vivo phenotype.** (*A*) Intestinal tissue sections from the colon of GP96^fl/fl^ (WT) and GP96^fl/fl^-Villin^creERT2^ (*Gp96* KO) mice collected on day 4 after first tamoxifen-injection stained for Ki67 (red). *Scale bar*: 50 μm. (*B*) Small intestinal tissue section of a GP96^fl/fl^-Villin^creERT2^ (*Gp96* KO) mouse collected on day 6 after first tamoxifen-injection. Stained for lysozyme (green) and Ki67 (red). *White arrows* mark Ki67-positive Paneth cells. *Scale bar*: 50 μm. (*C*) Western blot analysis on lysates from colonic epithelial cells collected on days 3 and 5 after first tamoxifen injection, normalized to β-actin. Graphs show representative Western blot images from each experimental group. N = 6 mice/group. (*D*) Western blot analysis on lysates from small intestinal epithelial cells collected on days 3 and 5 after first tamoxifen injection, normalized to β-actin. Graphs show representative Western blot images from each experimental group. N = 6 mice/group. (*E*) Quantitative reverse-transcription PCR analysis on colonic epithelial cells, normalized to glyceraldehyde-3-phosphate dehydrogenase (GAPDH) and treated WT control mice on days 1, 3, and 5. N = 2–7 mice/group. Significance was calculated using 2-way analysis of variance with a Tukey correction (reverse-transcription-PCR) or a Kruskal–Wallis test (1-way analysis of variance, nonparametric) (Western blot). *Bars* represent means with SD. *Asterisks* indicate significant differences, as follows ∗*P* ≤ .05, ∗∗*P* ≤ .01, ∗∗∗*P* ≤ .001, and ∗∗∗∗*P* ≤ .0001. mRNA, messenger RNA.

IEC-specific GP96 depletion in adult mice resulted in rapid weight loss within 6 days after the first tamoxifen injection (Figure 1*B*). On day 6, GP96-Villin^creERT2^ mice showed visible signs of intestinal inflammation, characterized by a general shortening of the colon and the small intestine, as well as a transparent and fragile-appearing small intestine and cecum wall (Figure 1*E*). This was in line with a significant, successive reduction of IEC numbers upon deletion of *Gp96*. In addition, the small intestine was filled with intestinal fluid, while solid food or fecal pellets were completely absent, indicating a severe defect of normal IEC function. H&E staining showed a deteriorated crypt–villus structure in the small intestine while the epithelial architecture in the colon was not affected to the same extent (Figure 1*G*). In accordance with these observations, proliferating cells at the crypt base were reduced markedly in the small intestine but not in the colon (Figures 3*A* and 4*A*), indicating that loss of GP96 affects ISC proliferation/function in a region-specific manner. Notably, ISC-specific deletion of *Gp96* in GP96-Lgr5^EGFP-IRES-creERT2^ mice did not result in a clinical and morphologic phenotype (Figure 2).

### Presence of GP96 Is Crucial for Maintaining the Small Intestinal Stem Cell Niche

We noticed that the reduction of proliferating cells in the small intestine was preceded by an almost-complete disappearance of GP96 in the villi compartment (Figure 1*F*), followed by a continuous loss of (Olfactomedin 4) OLFM4-expressing stem cells at the bottom of crypts (Figure 3*A*). In contrast, Paneth cells seemed to be more resistant to *Gp96* deletion (Figure 3*B*), which might be owing to high basal GP96 protein expression in these cells and the long half-life of GP96.^29^^,^^30^ However, on day 6 after tamoxifen-injection, the morphology and location of Paneth cells also were altered severely in GP96-Villin^creERT2^ mice (Figure 3*C*). A similar effect was observed for Mucin 2 (MUC2)-positive goblet cells (Figure 3*C*), and enlarged secretory cells lined the entire inside of the remaining crypts with a small number of Paneth cells that stained positive for Ki67 (Figure 4*B*). Because postmitotic Paneth cells normally do not proliferate,^31^ this observation indicates that some Paneth cells might have reacquired the proliferative capacity, possibly to compensate for the loss of OLFM4-expressing stem cells. In addition, some lysozyme-positive cells were found at unusual places, such as the blunted villi region. Real-time polymerase chain reaction (PCR) and Western blot analysis confirmed the continuous loss of stem cell marker, such as *Olfm4*/OLFM4, on the gene and protein expression level (Figures 3*D* and *E* and 4*E*). In addition, Western blot analysis showed a significantly reduced expression of the mature form of Wnt co-receptor LRP6 (Figures 3*E* and 4*C*), confirming the reported involvement of GP96 as chaperone in the LRP6 protein folding process.^6^^,^^9^ Thus, we then examined the effects of GP96 on Wnt and the closely intertwined Notch signaling pathway, which both directly regulate ISC maintenance. Indeed, the expression of Wnt target genes *Axin2* and *Cyclin-D1* started to decrease on days 3 and 5 (Figures 3*D* and 4*E*), respectively, in accord with the observed reduction of Ki67-positive, proliferating cells from day 3 on (Figure 3*A*). Interestingly, protein levels of nuclear β-catenin, indicative for active Wnt signaling, were lower on day 5, suggesting decreased Wnt activity (Figure 4*D*). In addition, we noted lower levels of NOTCH1 receptor and cleaved Notch intracellular domain, which indicates reduced Notch signaling activity (Figure 3*E*). In line with this trend, real-time PCR showed a decrease in the expression of Notch target gene *Hes1* (Figures 3*D* and 4*E*). Inhibition of Notch signaling has been shown to promote the differentiation of IECs into secretory cell types.^22^ However, in our mouse model we could not detect any significant increase in the expression of marker genes for secretory cells (*Dll1* and *Atoh1*), Paneth cells (*Lyz1*), goblet cells (*Muc2*), or enteroendocrine cells (*Chga*) (Figure 3*D*).

Taken together, these experiments show that depletion of GP96 leads to a consecutive loss of stem cells, proliferating cells, and, ultimately, degeneration of differentiated IECs, resulting in complete disruption of the small intestinal epithelial layer, ultimately, culminating in death of the mice (Figure 3*F*).

### GP96 Depletion Causes Loss of Stemness and Degeneration of Small Intestinal Organoids

**Figure 5:**
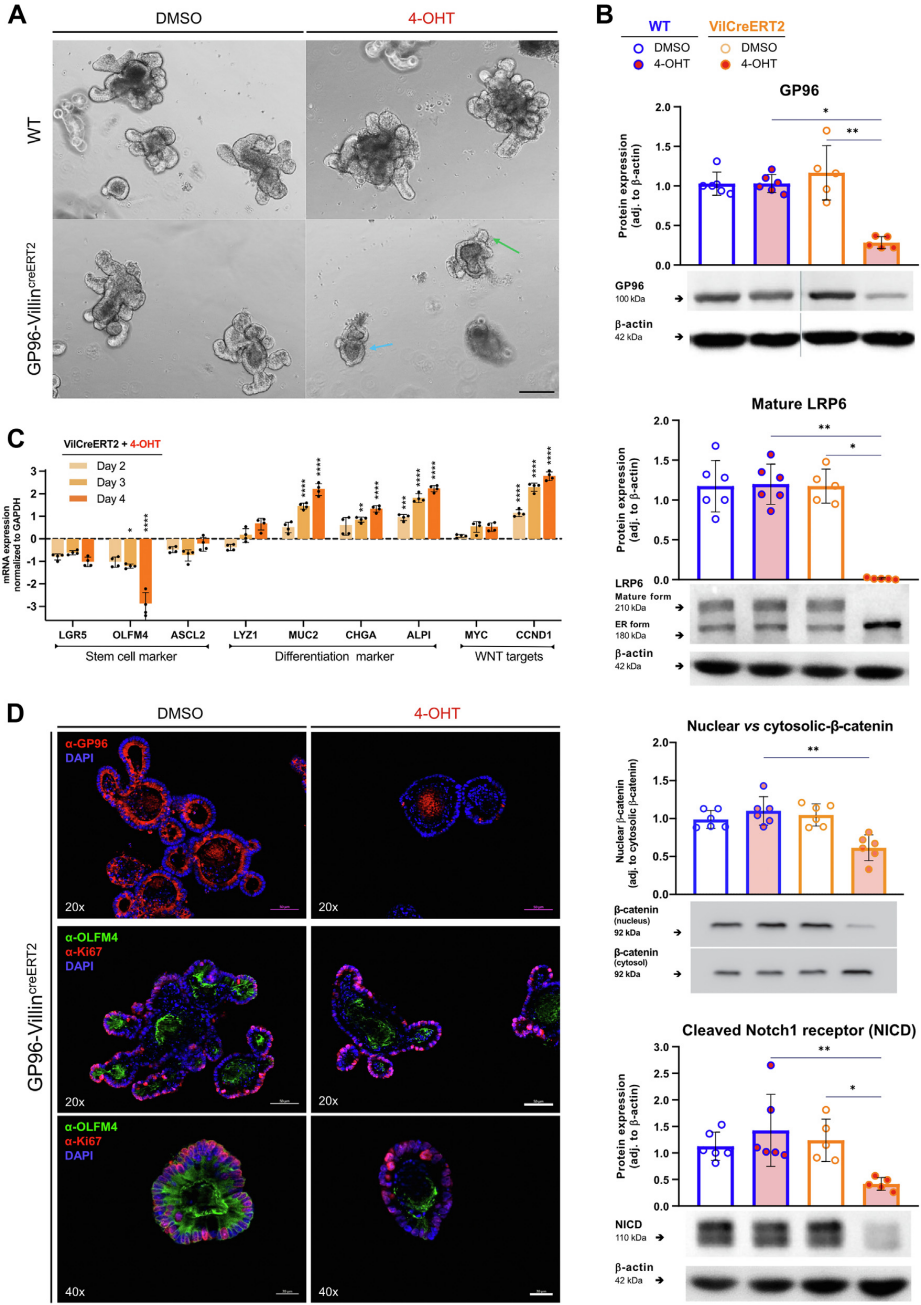
I**nduction of *Gp96* KO in small intestinal organoids reduces stemness and increases expression of differentiated cell type marker.** Organoids were generated from duodenal crypts of GP96^fl/fl^ (WT) and GP96-Villin^creERT2^ mice. Cre-mediated recombination was induced by adding 4-OHT (2 μmol/L) or an equal volume of dimethyl sulfoxide (DMSO) to the culture medium for 48 hours. (*A*) Light microscope images on day 4. Images are representative of 3 independent experiments. *Bluearrow* highlights immature spheroids with complete absence of normal crypt–villi structure, *green arrow* highlights budds. Original magnification, ×10. (*B*) Western blot analysis of cell lysates collected on day 4 after *Gp96* KO induction, normalized to β-actin. Representative protein bands from each experimental group are shown, originating from the same gel/blot (cropped regions are marked by *dividing lines*). (*C*) Quantitative reverse-transcription PCR analysis, normalized to glyceraldehyde-3-phosphate dehydrogenase (GAPDH) and 4-OHT–treated WT organoids collected on the same day. (*D*) GP96 (red) (*upper panels*), OLFM4 (green) and Ki67 (red) (*lower panels*) immunofluorescence on GP96-Villin^creERT2^ small intestinal organoids fixed on day 4 after *Gp96* deletion. *Scale bars*: 50 μm (×20) or 20 μm (×40). Statistics were performed using 2-way analysis of variance with a Tukey correction (reverse-transcription PCR) and by a Kruskal–Wallis test (1-way analysis of variance, nonparametric) with a post hoc correction for multiple comparisons using the Dunn test (Western blot). *Bars* represent means with SD. Biological replicates, N = 6. *Asterisks* indicate significant differences, as follows: ∗*P* ≤ .05, ∗∗*P* ≤ .01, ∗∗∗*P* ≤ .001, and ∗∗∗∗*P* ≤ .0001. adj, adjusted; DAPI, 4′,6-diamidino-2-phenylindole; mRNA, messenger RNA.

Given the severe phenotype of *Gp96* KO in GP96-Villin^creERT2^ mice, we generated small intestinal organoids from GP96-Villin^creERT2^ and control mice to further study the molecular pathways affected in IECs upon *Gp96* deletion. Consistent with our in vivo data from GP96-Villin^creERT2^ mice, deletion of *Gp96* by addition of (Z)-4-hydroxytamoxifen (4-OHT) to the organoid culture media significantly impacted organoid morphology. Compared with control organoids not expressing the Villin^creERT2^ construct, Villin^creERT2^ organoids were not able to develop the typical multilobed, crypt–villi structure and a higher abundance of immature spheroids were observed (Figure 5*A*, blue arrow). Of note, changes in the morphology of Villin^creERT2^ organoids only became visible 4 days after 4-OHT treatment (Figure 5*A*), which is consistent with the reported half-life of GP96 of 3–4 days.^29^^,^^30^ Indeed, Western blot analysis showed reduced but still detectable levels of GP96 4 days after *Gp96* KO induction (Figure 5*B*).

To detect and track changes in the gene expression that might provoke the morphologic changes of *Gp96* KO organoids, we isolated RNA on days 2, 3, and 4 after 4-OHT treatment. Consistent with the loss of stem cells in tamoxifen-treated GP96-Villin^creERT2^ mice, expression of the stem cell markers *Lgr5*, *Olfm4*, and *Ascl2* was reduced in GP96-Villin^creERT2^ organoids, with *Olfm4* showing the most pronounced reduction, followed by *Lgr5* (Figure 5*C*). In contrast, some differentiation markers (ie, the goblet cell marker *Muc2* and the enteroendocrine cell marker *Chga*) were enhanced. A similar trend was observed for the enterocyte marker *Alpi*. Interestingly, typical Wnt signaling target genes *Myc* and *Cyclin-D1* were not down-regulated significantly in organoids with *Gp96* deletion. On the contrary, messenger RNA levels of *Cyclin-D1* were increased, indicating either ongoing activation of the canonical Wnt/β-catenin pathway or the involvement of compensation mechanisms in response to impaired Wnt signaling. To further examine this effect, we performed Western blot analysis for LRP6 and active (nonphosphorylated) β-catenin. Although the levels of the mature form of Wnt co-receptor LRP6 were reduced significantly in *Gp96* KO organoids, the decrease in active β-catenin was only approximately 50% (Figure 5*B*), confirming reduced but ongoing Wnt/β-catenin activity. This observation was supported further by immunofluorescence staining for proliferation marker Ki67 (Figure 5*D*), which showed that proliferating cells still were present in *Gp96* KO organoids on day 4 despite the absence of GP96-positive IECs and the reduction of OLFM4-expressing stem cells. Taken together, gene and protein expression analysis of *Gp96* KO organoids showed a decrease, but not complete loss, of β-catenin activity, a steady reduction of typical stem cell markers, and a shift in the gene expression signature toward more differentiated IECs.

### Depletion of GP96 in ISCs Primarily Triggers a Greater Level of Differentiation but Not Apoptosis

**Figure 6:**
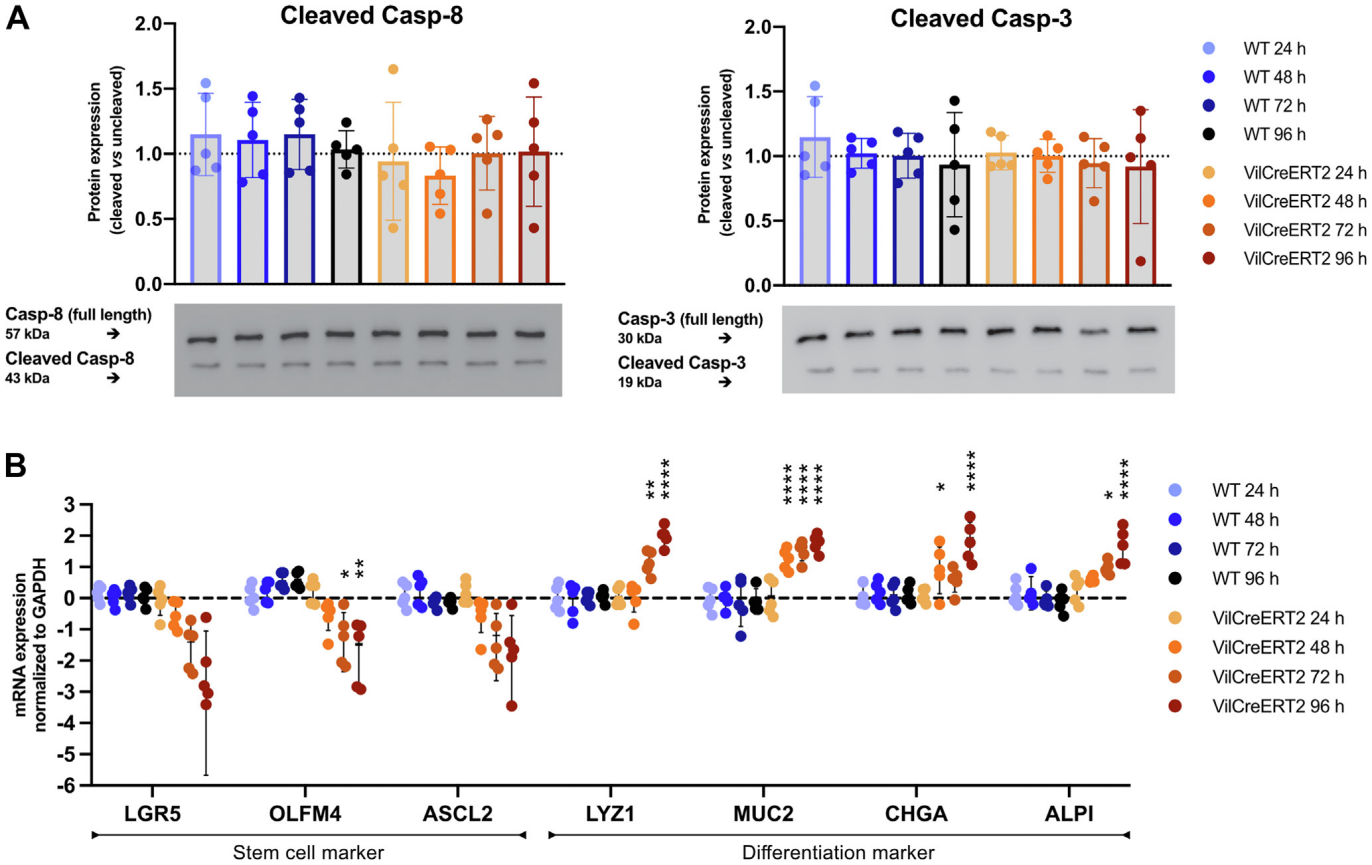
**GP96-deficiency in ISCs provokes premature differentiation into IEC subsets but not increased apoptosis.** ISCs were isolated from GP96^fl/fl^ (WT) and GP96-Villin^creERT2^ (VilCreERT2) mice. Cre-mediated recombination was induced by adding 4-OHT (1 μmol/L) to the culture medium. (*A*) Western blot analysis on ISC lysates collected 24, 48, 72, and 96 hours after treatment with 4-OHT. Graphs show representative Western blot images from each experimental group. (*B*) Quantitative reverse-transcription PCR analysis of relative messenger RNA (mRNA) expression, normalized to glyceraldehyde-3-phosphate dehydrogenase (GAPDH) and to WT control at 24 hours. Statistics were performed using 2-way analysis of variance (reverse-transcription PCR, WT vs VilCreERT2 at specific time points) and 1-way analysis of variance (Western blot) with a Tukey correction. *Bars* represent means with SD. Biological replicates N = 5. *Asterisks* indicate significant differences, as follows: ∗*P* ≤ .05, ∗∗*P* ≤ .01, , and ∗∗∗∗*P* ≤ .0001.

To date, the observed increase in differentiation markers upon deletion of *Gp96* in IECs could result from a loss of stemness or from enhanced apoptosis of GP96-deficient ISCs. To test whether GP96-deficient ISCs show increased apoptosis, we sorted stem cells from wild-type (WT) and GP96-Villin^creERT2^ mice based on expression of Epithelial cell adhesion molecule (EpCAM) and CD24. The cells then were incubated in 4-OHT–containing medium and analyzed for the apoptosis markers cleaved caspase-8 and cleaved caspase-3. No changes were observed for these 2 markers or upon measuring viability (Figure 6*A*). Although markers of stemness, such as *Lgr5*, *Olfm4*, and *Ascl2*, were reduced, markers for IEC differentiation were increased after 72 and 96 hours (Figure 6*B*).

These data indicate that GP96-deficient ISCs preferentially differentiate into more mature IEC subsets, rather than dying off owing to a lack of GP96.

### Combined Wnt and Notch Inhibition in WT Organoids Results in a Gene Expression Pattern Similar to That Observed in GP96-Deficient Organoids

**Figure 7:**
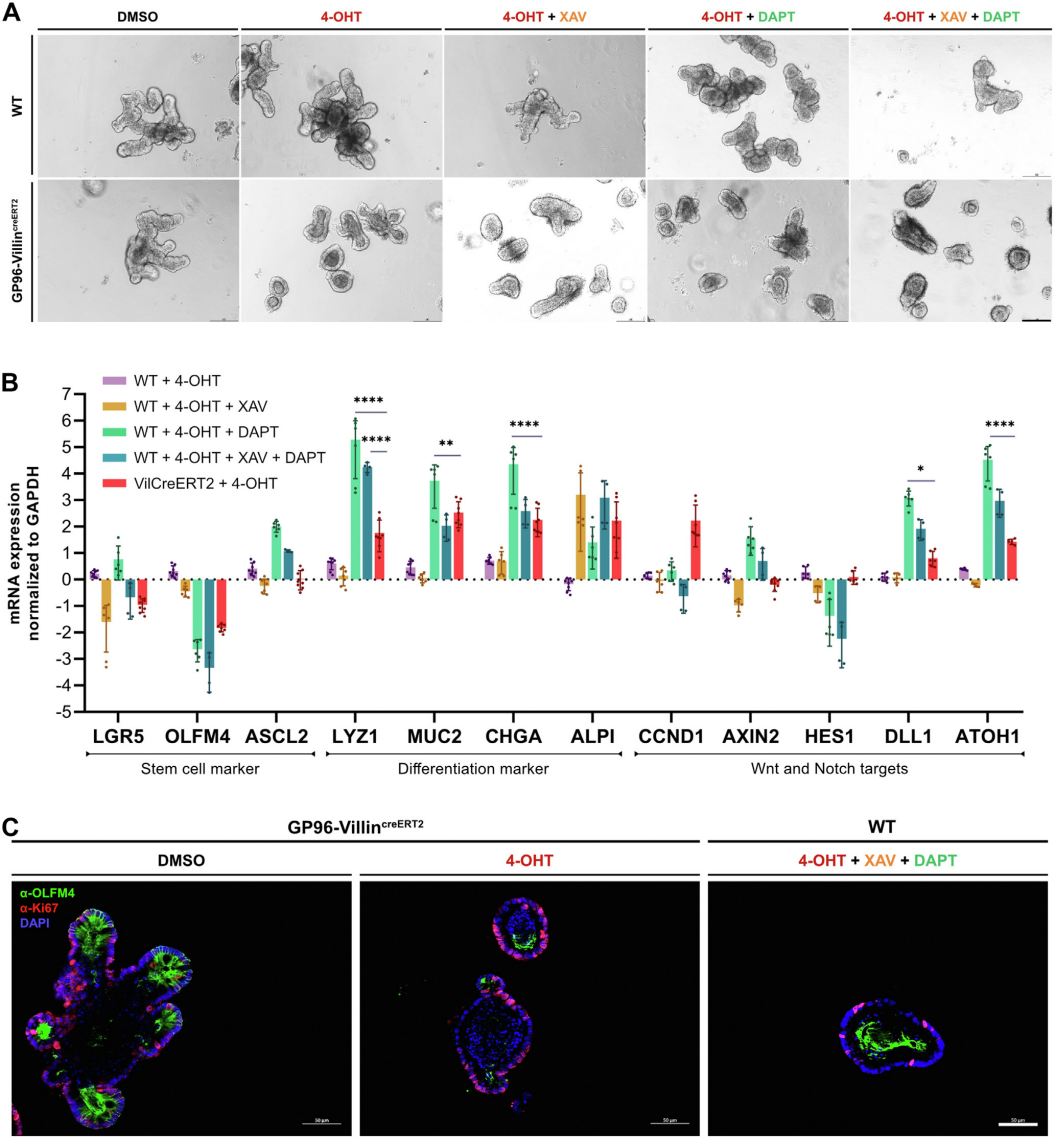
**Gene expression signature of Wnt- and Notch-inhibited WT organoids is similar to *Gp96* KO organoids.** Intestinal organoids were generated from jejunal crypts of GP96^fl/fl^ (WT) and GP96-Villin^creERT2^ (VilCreERT2) mice. Cre-mediated recombination was induced by adding 4-OHT (1 μmol/L) or an equal volume of dimethyl sulfoxide (DMSO) to the culture medium for 48 hours. Wnt signaling was inhibited with tankyrase inhibitor XAV939 (XAV, 10 μmol/L) and Notch signaling with γ-secretase inhibitor DAPT (10 μmol/L) for 96 hours starting on the day of 4-OHT treatment. (*A*) Representative images on day 4 (1 of 3 independent experiments). Original magnification, ×10. (*B*) Quantitative reverse-transcription PCR analysis of relative messenger RNA (mRNA) expression, normalized to glyceraldehyde-3-phosphate dehydrogenase (GAPDH) and DMSO-treated WT control organoids on day 4. (*C*) Immunofluorescence staining of organoids fixed on day 4 after *Gp96* KO induction (4-OHT) and Wnt (XAV)/Notch (DAPT) signaling inhibition, stained for OLFM4 (green) and Ki67 (red). *Scale bars*: 50 μm. Statistical analysis was performed using 2-way analysis of variance with the Dunnett method for multiple comparisons (reverse-transcription PCR). *Bars* represent means with SD. Biological replicates N = 3. *Asterisks* indicate significant differences, as follows: ∗*P* ≤ .05, ∗∗*P* ≤ .01, and ∗∗∗∗*P* ≤ .0001. DAPI, 4′,6-diamidino-2-phenylindole.

Because we observed defects in Notch and Wnt signaling in our GP96-Villin^creERT2^ mice, we tested whether single or combined Notch and Wnt inhibition in WT organoids can recapitulate the phenotype of tamoxifen-treated GP96-Villin^creERT2^ (*Gp96* KO) organoids. Indeed, Wnt- or Notch-inhibited WT organoids showed similar gene expression profiles as *Gp96* KO organoids: in both conditions, stem cell markers were reduced, whereas markers for differentiated cell types were up-regulated (Figure 7*B*). Of note, the combination of GP96 depletion plus Notch or Wnt inhibition led to an even more pronounced decrease of stem cell marker expression, which might explain the observed exacerbated morphologic phenotype of *Gp96* KO organoids treated with Wnt inhibitor (XAV939; XAV) and Notch inhibitor (N-[N-(3, 5-difluorophenacetyl)-l-alanyl]-s-phenylglycinet-butyl ester; DAPT) (Figure 7*A*). In line with these observations, Western blot analysis showed that GP96 depletion, similar to Notch inhibition, resulted in lower levels of the Notch intracellular domain (Figure 5*B*), indicating reduced Notch signaling activity and explaining the down-regulation of *Olfm4* and up-regulation of secretory cell type markers in *Gp96* KO and Notch-inhibited but not Wnt-inhibited organoids. However, even combined Wnt/Notch inhibition in WT organoids did not fully reflect the effect of *Gp96* deletion. In contrast, *Gp96* KO showed increased *Cyclin-D1* expression (Figure 7*B*) and depicted higher numbers of proliferating cells, as indicated by staining for the proliferation marker Ki67 (Figure 7*C*). Together, these results indicate that *Gp96* deletion directly or indirectly affects both Wnt and Notch signaling, and that defects in these pathways seem to be partially responsible for the defective regenerative potential of *Gp96* KO organoids.

### Combined Activation of Wnt and Notch Signaling Partially Restores Stem Cell Marker Expression in *Gp96* KO Organoids but Is Not Sufficient to Rescue Viability

**Figure 8:**
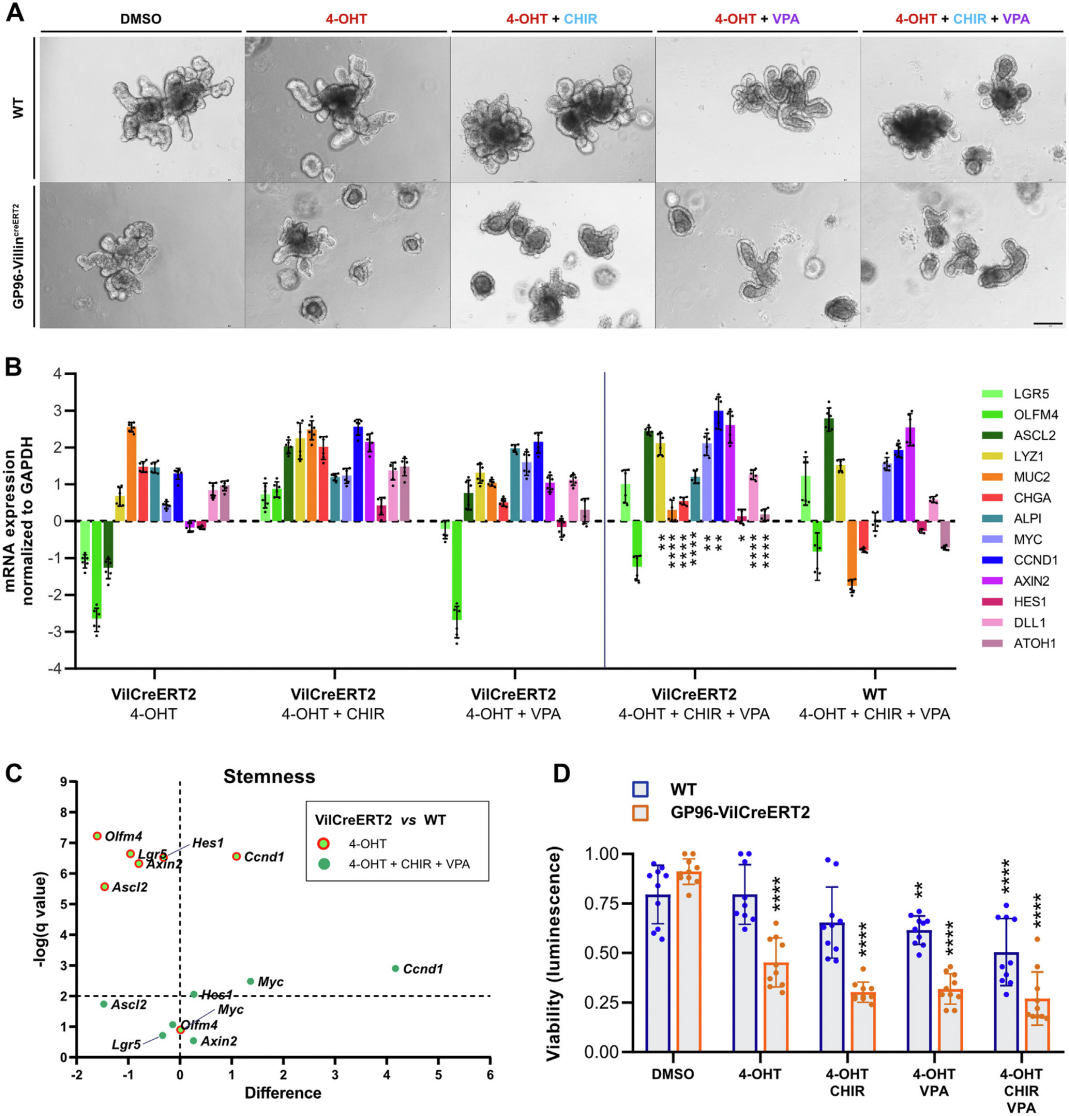
**Phenotype in *GP96*-deficient organoids cannot be rescued by activation of Wnt and Notch signaling.** Intestinal organoids were generated from jejunal crypts of GP96^fl/fl^ (WT) and GP96-Villin^creERT2^ (VilCreERT2) mice. Cre-mediated recombination was induced by adding 4-OHT (1 μmol/L) or an equal volume of DMSO to the culture medium for 48 hours. Two days after *Gp96* KO induction, Wnt signaling was activated with glykogen synthase kinase 3β inhibitor CHIR99021 (CHIR, 5 μmol/L) and Notch signaling with histone deacetylation inhibitor VPA (1 mmol/L) for 48 hours. (*A*) Representative live-cell images (3 independent experiments). Original magnification, ×10. (*B*) Quantitative reverse-transcription PCR analysis normalized to glyceraldehyde-3-phosphate dehydrogenase (GAPDH) and dimethyl sulfoxide (DMSO)-treated control organoids on day 4. (*C*) Volcano plot constructed by plotting on the x-axes the differences in gene expression (multiple *t* tests) between WT and VilCreERT2 organoids treated with 4-OHT or 4-OHT + CHIR + VPA. The negative log of the corresponding q value (significance) is shown on the y-axes. (*D*) Organoid viability relative to the highest value of the DMSO-treated control group, pooled data of 2 independent experiments. Statistics were performed using 2-way analysis of variance with the Dunnett method for multiple comparisons (reverse-transcription PCR, control group: VilCreERT2 + 4-OHT), multiple *t* test (volcano plot, control groups: VilCreERT2 + 4-OHT, VilCreERT2 + 4-OHT + CHIR + VPA) or by 2-way analysis of variance with a Sidak correction for multiple comparisons (viability assay, control groups: WT + DMSO, VilCreERT2 + DMSO). *Bars* represent means with SD. Biological replicates N = 3. *Asterisks* indicate significant differences, as follows: ∗*P* ≤ .05, ∗∗*P* ≤ .01, and ∗∗∗∗*P* ≤ .0001. mRNA, messenger RNA.

To further evaluate whether the effect of GP96 depletion resulted from impaired Wnt/β-catenin and Notch signaling, we enhanced canonical Wnt signaling using a selective glykogen synthase kinase 3β inhibitor (CHIR99021) and simultaneously applied the selective histone deacetylation inhibitor valproic acid (VPA) that is commonly used as a Notch signaling activator. Reverse-transcription PCR analysis 2 days after glykogen synthase kinase 3β inhibition confirmed increased Wnt signaling in CHIR-treated WT organoids (Figure 8*B*). Interestingly, although the treatment of *Gp96* KO organoids with CHIR + VPA was able to slightly augment the gene expression of the stemness markers *Lgr5*, *Olfm4*, and *Axin2*, and the Paneth cell marker *Lyz1* (Figure 8*B* and *C*), neither single treatment nor the combination of Wnt and Notch activation was able to restore viability in *Gp96* KO organoids. Moreover, quantitative assessment of organoid growth/viability showed that the overall viability was reduced even further upon treatment with CHIR99021 and VPA (Figure 8*A* and *D*). These results might indicate an essential role of GP96 in Wnt and Notch signaling that is required for organoid survival, but because the inhibitors also affect other signaling pathways, it is possible that other factors are involved in mediating the reduced survival of GP96-deficient organoids and, owing to down-regulation of Wnt and Notch signaling, pharmacologic activation of these pathways might be compromised in GP96-deficient organoids.

### GP96 Deficiency Leads to Increased ER Stress and Up-Regulation of ER Chaperone Immunoglobulin Binding Protein (BIP)/Glucose-Regulated Protein 78

**Figure 9:**
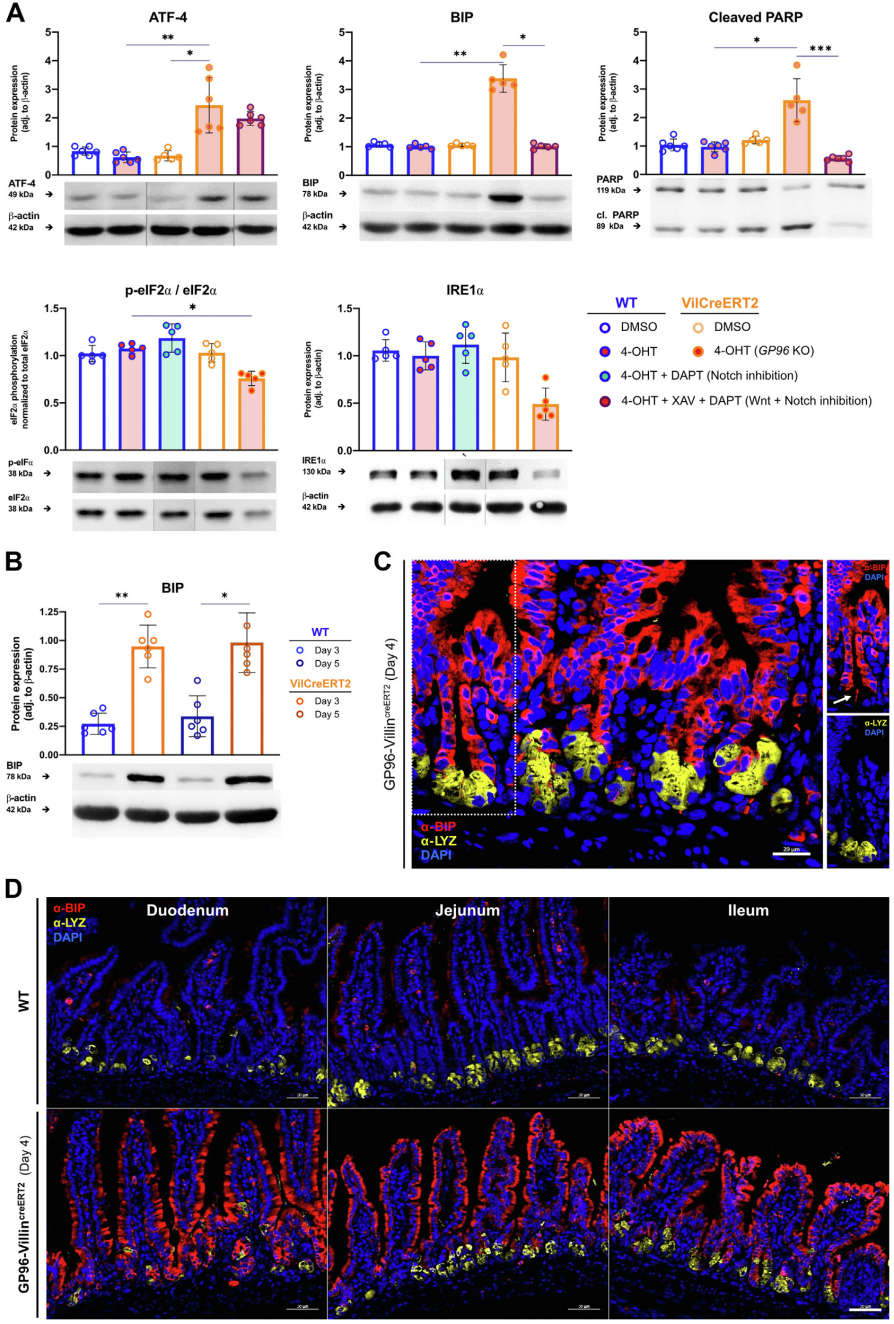
**Depletion of GP96 induces ER stress and up-regulates BIP expression in GP96-Villin**^**creERT2**^**organoids and mice.** (*A*) Intestinal organoids were generated from jejunal crypts of GP96^fl/fl^ (WT) and GP96-Villin^creERT2^ (VilCreERT2) mice. Cre-mediated recombination was induced by adding 4-OHT (1 μmol/L) or an equal volume of dimethyl sulfoxide (DMSO) to the culture medium for 48 hours. Wnt signaling was inhibited with tankyrase inhibitor XAV939 (XAV, 10 μmol/L) and Notch signaling with γ-secretase inhibitor DAPT (10 μmol/L) for 48 hours starting on the day of 4-OHT treatment. (*A*) On day 4 after *Gp96* KO induction, cells were collected for protein isolation and lysates used for Western blot analysis, normalized to β-actin. Representative protein bands from each experimental group are shown, originating from the same gel/blot (marked by *dividing lines*). (*B*) Western blot analysis on lysates from small intestinal epithelial cells collected on days 3 and 5 after first tamoxifen injection, normalized to β-actin. Graphs show representative Western blot images from each experimental group. (*C*) Small intestinal tissue section of a GP96-Villin^creERT2^ (*Gp96* KO) mouse collected and fixed on day 4 after first tamoxifen injection was stained for BIP (red) and lysozyme (yellow). *White arrow* marks BIP-positive ISC-like cell. *Scale bar*: 20 μm. (*D*) Small intestinal tissue sections of GP96^fl/fl^ (WT) and GP96-Villin^creERT2^ (*Gp96* KO) mice collected and fixed on day 4 after first tamoxifen injection were stained for BIP (red) and lysozyme (yellow). *Scale bars*: 50 μm. Significance was calculated using a Kruskal–Wallis test (1-way analysis of variance, nonparametric) and a post hoc correction for multiple comparisons with a Dunn test. *Bars* represent means with SD. Biological replicates N = 3 (organoids) and N = 5–6 (mice). *Asterisks* indicate significant differences, as follows: ∗*P* ≤ .05, ∗∗*P* ≤ .01, and ∗∗∗*P* ≤ .001. DAPI, 4′,6-diamidino-2-phenylindole; eIF2α, Eukaryotic translation initiation factor 2α; PARP, Poly (ADP-ribose) polymerase.

GP96 has been described as an ER chaperone and our in vivo observations showed morphologic alterations of Paneth and goblet cells, which might result from secretory ER stress. In line with this, protein levels of the main ER stress–induced transcription factor Activating Transcription Factor 4 (ATF-4) and the ER chaperone glucose-regulated protein (GRP)78, also known as Immunoglobulin Binding Protein (BIP), were increased significantly in *Gp96* KO organoids on day 4, while early ER stress markers such as phospho-eIF2α (Eukaryotic Translation Initiation Factor 2A) and IRE1α (Inositol-requiring enzyme-1a) were down-regulated, likely resulting from a compensatory response (Figure 9*A*). Unresolved ER stress promotes apoptosis, which can be measured by an increased ratio of cleaved to uncleaved PARP (Poly (ADP-ribose) polymerase).^32^ Indeed, *Gp96* KO organoids showed increased levels of cleaved PARP, especially when compared with Wnt- and Notch-inhibited WT organoids, despite similar expression of the ER stress marker ATF-4. Likewise, we detected a significant up-regulation of BIP in the intestinal epithelium of GP96-Villin^creERT2^ mice (Figure 9*B*). To localize BIP expression within the epithelium, we performed immunofluorescence co-staining for BIP and Paneth cells (lysozyme [LYZ]). In the crypt region and subsequent TA zone, highly increased expression of BIP was observed on day 3 after the first tamoxifen injection. Strikingly, a pronounced up-regulation of BIP also was visible in slender cells adjacent to Paneth cells, most likely remaining ISCs (Figure 9*C*). ER stress is typically absent in ISCs and its activation has been shown to trigger the transition from self-renewal to the TA state. On day 4, BIP-positive cells extended throughout the length of the crypt–villus axis (Figure 9*D*), suggesting that depletion of GP96 affects not only protein homeostasis in ISCs, but also in differentiated IECs. Interestingly, increased BIP expression was co-localized with LYZ-positive cells only in rare instances. This suggests that Paneth cells, despite high pressure for protein folding capacity, have not adopted a compensatory response at the time points analyzed.

### GP96 Is Enriched in Paneth Cells at Crypts of Mouse and Human Small Intestine

**Figure 10:**
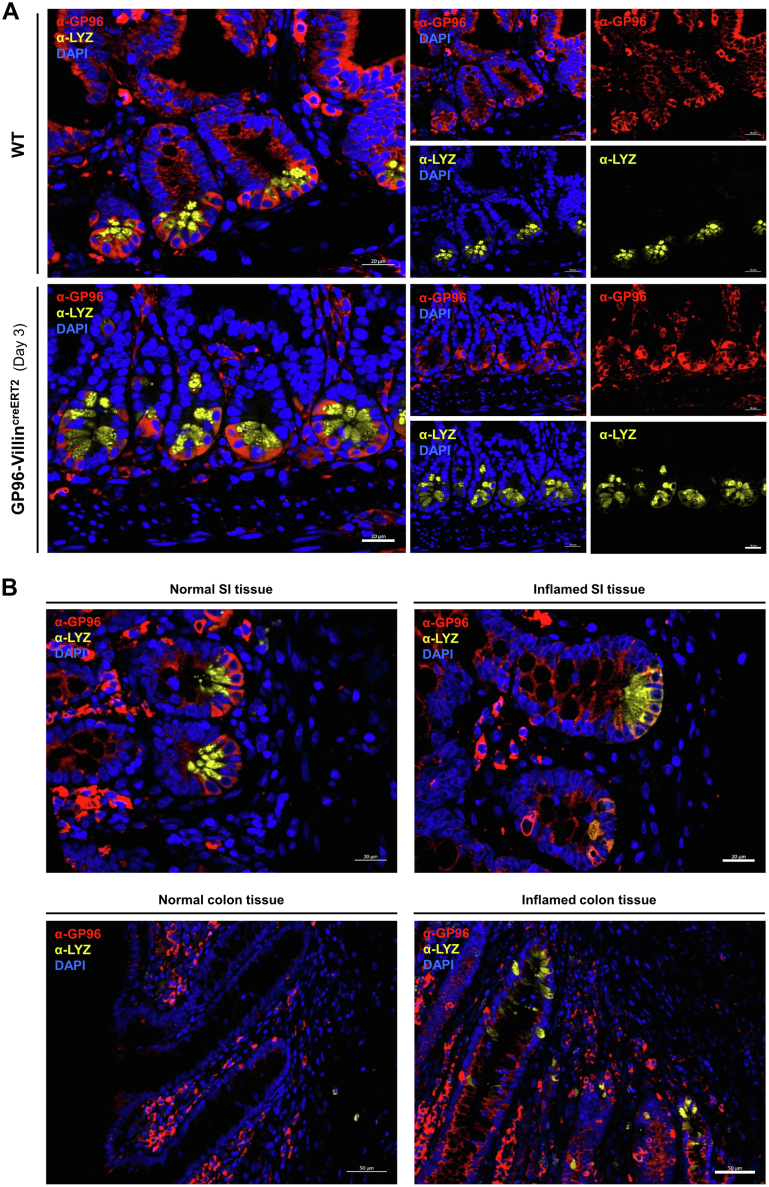
**GP96 is highly expressed in lysozyme-positive Paneth cells located in the crypt region.** Small intestinal (SI) tissue sections of (*A*) GP96^fl/fl^ (WT) and GP96-Villin^creERT2^ mice 3 days after *Gp96* deletion and (*B*) SI and colonic tissue of IBD patients were co-stained for lysozyme (yellow) and GP96 (red). *Scale bars*: 20 μm (*A* and *B*, SI tissue), and 50 μm (*B*, colon tissue). DAPI, 4′,6-diamidino-2-phenylindole.

To examine whether remaining functional GP96 could account for the observed lower BIP expression in Paneth cells of *Gp96* KO mice, we conducted immunofluorescence co-staining for LYZ and GP96. Indeed, our analysis on small intestinal tissue showed that LYZ-positive cells were largely overlapping with the few remaining GP96-positive cells in the crypt region of *Gp96* KO mice (Figure 10*A*). A generally high abundance of GP96 in Paneth cells also was observed in healthy GP96^fl/fl^ control mice and human intestinal tissue, whereas in other IECs GP96 was hardly detectable (Figure 10*B*). This indicates an increased demand for GP96 in Paneth cells, where it might support the high-protein folding activity in the ER. Interestingly, we also observed Paneth cells with marked GP96 expression in inflamed colonic tissue (Figure 10*B*, lower panel), which might be the result of an adaptive mechanism upon persistent intestinal inflammation.

**Figure 11:**
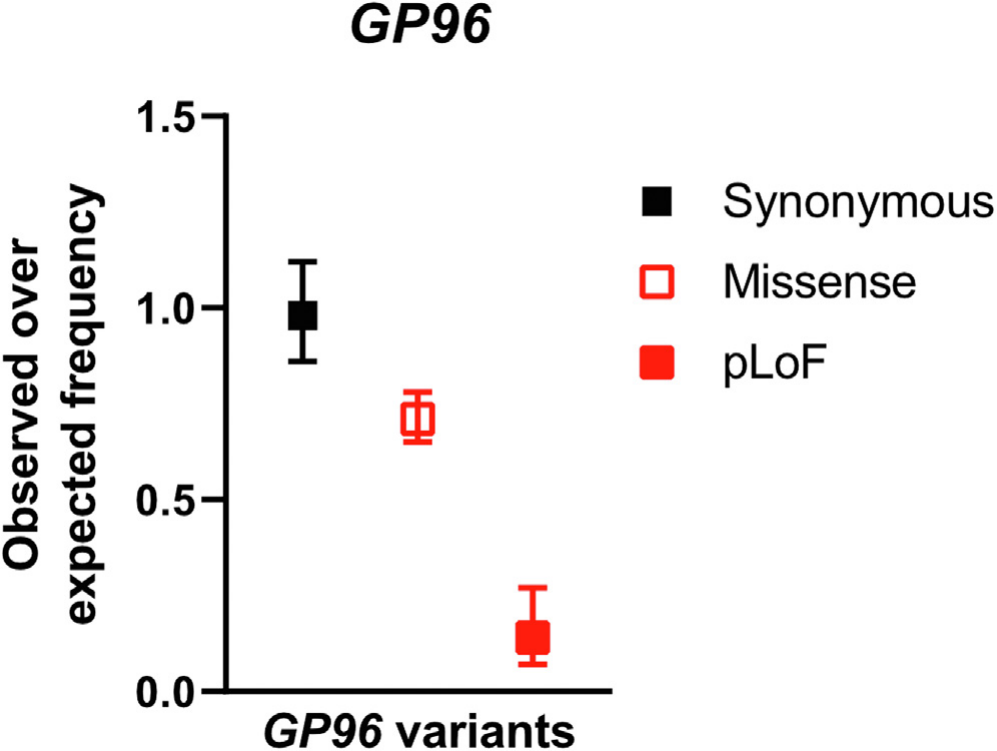
**Predicted loss-of-function (pLoF) *Gp96* variants are under-represented in the human population.** The observed-to-expected ratio of synonymous, missense, and pLoF variants in the *Gp96* gene are shown based on the Genome Aggregation Database (https://gnomad.broadinstitute.org).

Taken together, and in line with previous publications, these results show that GP96 depletion results in increased ER stress and that Paneth cells, which are especially vulnerable to ER stress owing to their secretory phenotype, express especially high levels of GP96. In accord with our in vivo observation that GP96 loss cannot be compensated by other proteins, resulting in lethality in mice, analysis of genetic variants found in human subjects within the gene locus encoding GP96 showed a significant under-representation of predicted loss-of-function variants (Figure 11). These results further underline the essential role of GP96 in mouse as well as human physiology.

## Discussion

Intestinal architecture and barrier integrity are maintained through continuous replacement of IECs by proliferating progenitors that originate from multipotent ISCs, which are located at the bottom of the crypt. In the ISC niche, complex interactions between multiple signaling pathways are required to ensure self-renewal capacity and to balance proliferation and differentiation. Here, we report that the molecular chaperone GP96 is directly or indirectly involved in 2 pathways especially critical for the regulation of ISC maintenance and IEC differentiation, namely Wnt and Notch signaling. In addition, depletion of GP96 resulted in marked ER stress, a process that further modulates the balance between self-renewal, differentiation, and apoptosis of IECs. Using the GP96-Villin^creERT2^ mouse model and intestinal organoid technology we showed that deletion of *Gp96* in IECs resulted in loss of ISCs and proliferating IECs, morphologic alterations of the remaining epithelial cells, and culminated in complete eradication of the intestinal epithelium. We show that these effects resulted from a combination of compromised Wnt and Notch signaling paired with excessive ER stress, and not only a mere consequence of disturbed Wnt signaling as previously suggested.^33^ This clearly indicates that GP96 is indispensable for intestinal epithelial homeostasis by enabling proper function of several crucial pathways involved in IEC and ISC homeostasis.

Our findings are consistent with a previous report showing that GP96 acts as a molecular chaperone for the Wnt co-receptor LRP6.^9^ However, although this report focused on Wnt signaling to explain impaired IEC function upon deletion of *Gp96*, we here show that multiple pathways contribute to this phenotype. Although protein expression of the mature form of LRP6 was reduced significantly in IECs from *Gp96* KO mice and organoids, we did not observe a complete reduction in active/nuclear β-catenin. Moreover, *Gp96* KO organoids showed increased levels of *Ccnd1*, suggesting at least some residual Wnt signaling activity despite reduced LRP6 expression. Up-regulation of Wnt signaling in facultative stem cells, in particular increased expression of *Ascl2*, has been shown to be part of a compensatory response to replace impaired stem cells and restore homeostasis upon tissue injury.^31^^,^^34^ Indeed, we observed several enlarged Paneth cells that stained positive for the proliferation marker Ki67, indicative of reacquired proliferative and self-renewal capacity. In the long term, however, those cells were not capable of counteracting the rapid onset of epithelial integrity loss. Notably, restoration of the intestinal stem cell niche upon damage requires not only continued Wnt signaling, but also the induction of Notch signaling to promote permissive conditions for regeneration.^35^ Thus, impaired activation of both of these pathways upon *Gp96* deletion might explain the complete inability to regenerate and the failure of Paneth cells to compensate for ISC loss. Interestingly, although a trend to decreased stemness also was observed in colonic IECs, the colon did not show any morphologic alterations at the analyzed time points, which is in sharp contrast to the severely affected small intestinal epithelium. This higher resilience to perturbations could be explained by either the slower turnover time of the colonic epithelium^1^ or might be linked to the different cellular composition. In fact, higher basal expression of *Lgr5* in the colon^36^ may confer enhanced resistance to damage, DNA repair, and growth advantage on colonic ISCs and thereby possibly also explain the increased prevalence of cancer in the colon when compared with the small intestine.

In addition to the previously reported involvement of GP96 in the Wnt/β-catenin signaling pathway, our data indicate a blockade of Notch signaling (ie, reduced levels of Notch1/cleaved Notch1) and an increase of secretory cell markers.^22^^,^^37^ In contrast, the Paneth cell marker *Lyz1* remained unchanged. In addition to absence of Notch stimulation, Paneth cell maturation requires the Wnt downstream transcription factor SRY-Box Transcription Factor 9 (Sox9) and the Wnt-induced expression of the Matrix Metallopeptidase 7 (MMP-7)/cryptdin program.^38^^,^^39^ Thus, the observed gene expression signature upon *Gp96* KO may be the result of combined Notch and Wnt inhibition. Previously described opposing activities of the Wnt and Notch pathway^25^ might explain the modest increase in secretory cell markers in GP96-deficient IECs despite impaired Wnt or Notch signaling, respectively. Notably, however, in vitro rescue of the phenotype by enhancing Wnt and Notch signaling simultaneously in *Gp96* KO organoids was not successful. These observations show that loss of viability owing to GP96 depletion is not solely the result of impaired Wnt and Notch signaling.

In line with this, GP96 also has been attributed a housekeeping role in ER homeostasis by reducing ER stress and ER stress–induced cell death.^8^ Indeed, we observed an up-regulation of the ER stress marker GRP78/BIP. It has been shown that BIP is a key regulator of the unfolded protein response (UPR) and its expression is increased in the transition of stem cells to TA cells.^28^ Because stem cell loss in our model was accompanied by an increase in BIP expression, it is conceivable that activation of the UPR response resulting from GP96 depletion promotes ISC differentiation. Of note, depletion of BIP, analogous to GP96, has been shown to result in loss of self-renewal capacity.^28^^,^^40^ Despite the overlapping phenotypes, GP96 and BIP seem to play distinct roles in the epithelium. BIP is expressed primarily in TA and differentiated cells in the villus regions,^28^ whereas GP96 was most abundant in Paneth cells, where it might be required to cope with the high demand of protein synthesis. Increased GP96 expression in Paneth cells, coupled with a long turnover time, might explain the higher resistance of Paneth cells to GP96 depletion and their delayed up-regulation of BIP upon *Gp96* deletion compared with other IEC types.

Notably, additional surface proteins, including in particular Toll-like receptors and integrins,^8^ also might be affected by the loss of GP96. In this way, GP96 loss additionally may compromise immunologically relevant pathways in our in vivo model. Although Toll-like receptors on IECs are responsible for direct recognition of pathogenic microbes and mounting defense programs,^41^ integrins are required for proper crypt–villus compartmentalization by regulating adhesion, migration, and proliferation of IECs.^42^, ^43^, ^44^ Because we observed morphologic alterations and crypt degeneration in *Gp96* KO mice it is possible that impaired integrin signaling and reduced engagement with the extracellular matrix also contribute to the breakdown of the epithelial barrier integrity.

It is noteworthy that, similar to the murine intestine, the expression of GP96 in human intestinal epithelium was highest Paneth cells. Interestingly, we also detected GP96 and LYZ-positive cells in the inflamed colonic epithelium of IBD patients. Paneth cells normally only are detected in low numbers in the large intestine, but their numbers can be increased in pathologic states such as IBD.^45^ This observation further highlights the important role of GP96 in epithelial regeneration, but additional analysis will be necessary to characterize the role of GP96 in Paneth cells. Because Paneth cells, which can regain ISC identity in response to injury,^31^ express increased levels of GP96, and GP96 is highly relevant for maintaining a normal ISC balance, GP96 also might be a crucial factor for controlling the plasticity of IEC subtypes. This hypothesis is supported by our observation that ISC-specific GP96 depletion does not affect epithelial integrity, suggesting a replacement of *Gp96* KO ISCs by the remaining GP96-expressing IECs. In GP96-Villin^creERT2^ mice with epithelium-wide loss of GP96, however, this de-differentiation capacity of the otherwise highly plastic IECs appears to be severely compromised, preventing a successful regeneration of the intestinal epithelium. Follow-up analysis will require IEC-subtype–specific Cre-driver mouse lines, ideally in combination with reporter mouse models, which will additionally allow live cell imaging and cell lineage tracing. Consequences of altered GP96 abundance might be IEC type–specific and the performance of single-cell RNA sequencing analysis will be necessary to delineate the role of GP96 in specific IEC populations. Given the increasing interest in the mechanisms behind the remarkable plasticity of the intestinal epithelium, it is worthwhile to further explore how GP96 contributes to the self-renewal capacity of the damaged intestinal epithelium, which also might open doors to new therapeutic opportunities for diseases such as IBD.

To conclude, we here show that *Gp96* deletion in IECs affects not only epithelial integrity via compromised Wnt signaling in ISCs, but that it interferes with the balanced interplay of multiple signaling pathways and thereby provokes premature differentiation and ultimate cell death via UPR response. Consequently, the capacity of IECs to support the stem cell niche and to regain stem cell properties upon stem cell loss is severely compromised. Our work indicates that the deletion of *Gp96* has much more detrimental effects than Wnt inhibition alone. Thus, our study clearly shows a fundamental role of GP96 in intestinal epithelial homeostasis and highlights the interdependency of Wnt and Notch signaling, which has important implications for therapeutic targeting of those pathways.

## Material and Methods

### Materials

Chemicals, peptides, and recombinant proteins used in this study are listed in Table 1.

**Table 1:** Chemicals, Peptides, and Recombinant Proteins

Chemicals, peptides**,** and recombinant proteins	Source	Identifier	Concentration
(Z)-4-Hydroxytamoxifen	Sigma	T176	1–2 μmol/L
Advanced DMEM/F12 with L-glutamine (2.5 mmol/L), phenol red, HEPES (15 mmol/L)	Thermo Fisher Scientific	11330057	
BSA	Pan Biotech	PANP06-1391500	
Cell recovery solution	Corning	354253	
CHIR99021	Tocris	4423/10	5 μmol/L
cOmplete, Mini Protease Inhibitor Cocktail	Sigma/Roche	11836153001	
DAKO Target Retrieval Solution pH 6.0	Agilent/Dako	S169984-2	
Dako Fluorescent Mounting Medium	Agilent/Dako	S302380	
4',6-diamidino-2-phenylindole	Invitrogen	D1306	1:1000
DAPT	Tocris	2634	10 μmol/L
Dissolvent dimethyl sulfoxide	Sigma	41640	
Fetal bovine serum	Pan Biotech	P30-3602	
Gentle cell dissociation reagent	Stemcell Technologies	07174	
GoTaq G2 Hot Start Colorless Master Mix	Promega	M7433	
HBSS	Sigma	H2387	
Histoclear	Brunschwig	HS-200	
IntestiCult Organoid Growth Medium	Stemcell Technologies	06005	
Matrigel Corning growth factor reduced basement membrane matrix, phenol red-free	Corning	FAL356231	
M-PER lysis buffer	Thermo Fisher Scientific	78505	
Normal goat serum	Agilent/Dako	X0907	5%–10%
NuPAGE 4× LDS sample buffer	Thermo Fisher Scientific	NP0007	
Penicillin-streptomycin	Thermo Fisher Scientific	15140122	50 μg/mL
Pertex	Biosystems Switzerland AG	41-4012-00	
Tamoxifen	Sigma	T5648	1 mg/mouse
TaqMan Fast Universal PCR Master Mix (2×), no AmpErase UNG	Thermo Fisher Scientific	4367846	
Valproic acid, sodium salt	Tocris	2815	1 mmol/L
WesternBright ECL horseradish peroxidase substrate	Advansta	K-12045-D50	
XAV939	Abcam	ab120897	10 μmol/L
Y-27632	Stemcell Technologies	72302	10 μmol/L

DAPI, 4′,6-diamidino-2-phenylindole; DAPT, N-[N-(3, 5-difluorophenacetyl)-l-alanyl]-s-phenylglycinet-butyl ester; DMEM, Dulbecco’s modified Eagle medium; HRP, horseradish peroxidase.

### Animals

All animal procedures were conducted in accordance with the Swiss animal welfare legislation and approved by the Cantonal Veterinary Office (Zurich, Switzerland) (approval 021/2018). Animals were housed under specific pathogen-free conditions and provided with food and water ad libitum. Experiments were performed in 10- to 13-week-old mice, and crypts were isolated from 8- to 17-week-old mice. Littermate controls were used for all experiments. The investigators were not blinded to allocation while performing the experiment.

Mice with an inducible *Gp96* deletion in intestinal epithelial cells were generated by crossing mice with a loxP flanked *GP96* gene^46^ with mice expressing the Cre-ERT2 construct under the *Villin* (Villin^creERT2^ mice; Jackson Laboratories) promoter. To induce Cre-mediated deletion of the *GP96* allele, tamoxifen (T5648; Sigma) dissolved in peanut oil/96% ethanol (20:1) was injected (1 mg/mouse) intraperitoneally for 5 consecutive days. Day 0 was counted as the day of the first injection.

### Tissue Collection, H&E, and Immunofluorescence Staining

For histology, intestinal tissue from duodenum, jejunum, ileum, and proximal and distal parts of the colon were collected at the indicated time points after tamoxifen administration. Tissues were flushed with ice-cold phosphate-buffered saline (PBS), opened longitudinally, and 1-cm sections were fixed in 4% formalin buffer before embedding in paraffin, sectioning into 5-μm sections, deparaffinization with Histoclear (HS-200; Brunschwig), and rehydration with descending ethanol series. For H&E staining, standard methods were used and slides were mounted with Pertex (41-4012-00; Biosystems Switzerland AG). For immunofluorescence staining, slides were rehydrated, immersed in antigen retrieval solution (pH 6.0, S169984-2; DAKO), boiled in a water bath at 97°C for 30 minutes, and blocked with blocking buffer (PBS pH 7.2, 5% normal goat serum, 5% bovine serum albumin [BSA]) for at least 1 hour at room temperature. After blocking, slides were incubated with primary antibodies diluted in antibody buffer (PBS pH 7.2, 5% normal goat serum, 1% BSA) at 4°C overnight. The next day, secondary antibodies were added for 2 hours together with 20 μg/mL 4′,6-diamidino-2-phenylindole (D1306; Invitrogen) and slides were mounted with Dako Fluorescent Mounting Medium (S302380). Detailed information about antibodies and applied dilutions can be found in Table 2. Images were collected using a ZEISS Axio Imager.Z2 microscope equipped with a ZEISS mono and color Axiocam 503 and a ZEISS ApoTome.2 for optical sectioning. Deconvolution on the collected raw data files was conducted with ZEISS ZEN 2.6 software using a preselected set of default parameters. The deconvoluted images were processed further with ImageJ (National Institutes of Health).

**Table 2:** Antibodies

Antibodies	Source	Identifier	Application	Dilution
Goat anti-rabbit IgG (H+L) cross-adsorbed secondary antibody, Alexa Fluor 488	Invitrogen	A-11008	IF	1:500
Goat anti-rabbit IgG (H+L) cross-adsorbed secondary antibody, Alexa Fluor 647	Invitrogen	A-21244	IF	1:500
Goat anti-rabbit IgG secondary antibody, HRP linked	Cell Signaling	7074	WB	1:1000
Goat anti-rat IgG (H+L) cross-adsorbed secondary antibody, Alexa Fluor 546	Invitrogen	A-11081	IF	1:500
Horse anti-mouse IgG secondary antibody, HRP linked	Cell Signaling	7076	WB	1:1000
Mouse anti-actin (C4)	Chemicon International	MAB1501	WB	1:1000
Rabbit anti-ATF4 (D4B8)	Cell Signaling	11815S	WB	1:1000
Rabbit anti-BiP (C50B12)	Cell Signaling	3177	WB	1:1000
Rabbit anti-CHOP (L63F7)	Cell Signaling	2895	WB	1:1000
Rabbit anti–chromogranin A	Novus Biologicals	NB120-15160	IF	1:200
Rabbit anti-cleaved Notch1 (Val1744) (D3B8)	Cell Signaling	4147	WB	1:1000
Rabbit anti–E-cadherin (24E10)	Cell Signaling	3195	IF	1:200
Rabbit anti–E-cadherin (24E10) (AlexaFluor-594)	Cell Signaling	7687	IF	1:50
Rabbit anti-eIF2α (D3D7) XP	Cell Signaling	5324	WB	1:1000
Rabbit anti-GP96/GPR94 (D6X2Q) XP	Cell Signaling	20292	IF/WB	1:100/1:1000
Rabbit anti-LRP6 (C47E12)	Cell Signaling	3395	WB	1:1000
Rabbit antilysozyme (EPR2994[2])	Abcam	Ab108508	IF	1:250
Rabbit antilysozyme (DyLight-550)	Novus Biologicals	NBP2-61118R	IF	1:100
Rabbit anti-MUC2	Novus Biologicals	NBP1-31231	IF	1:500
Rabbit anti-nonphospho (active) β-catenin (D2U8Y)	Cell Signaling	19807	IF	1:2500
Rabbit anti-Notch1 (D1E11)	Cell Signaling	3608	WB	1:1000
Rabbit anti-OLFM4 (D6Y5A) XP	Cell Signaling	39141	IF / WB	1:400 / 1:1000
Rabbit anti-PARP	Cell Signaling	9542	WB	1:1000
Rabbit anti–phospho-eIF2α (Ser51) (119A11)	Cell Signaling	3597	WB	1:1000
Rat anti-Ki67 (SolA15)	Invitrogen	14-5698-80	IF	1:100

CHOP, CCAAT-enhancer-binding protein homologous protein; eIF2α, Eukaryotic translation initiation factor 2α; IF, immunofluorescence; H+L, heavy and light; HRP, horseradish peroxidase; MUC2, Mucin 2; WB, Western blot.

### IEC Isolation

IECs were isolated as previously described,^47^ with some modifications. Briefly, freshly isolated and washed intestines were cut into 5-mm pieces and stored in ice-cold Hank’s balanced salt solution (HBSS) (pH 7.4, H2387; Sigma) supplemented with 2% fetal calf serum. Subsequently, tissue pieces were incubated in ice-cold HBSS, 3 mmol/L EDTA, and 1.5 mmol/L dithiothreitol for 20 minutes, followed by a second incubation step in HBSS and 3 mmol/L EDTA for 15 minutes on a shaker at 200 rpm at 37°C. After EDTA chelation, the supernatant was discarded, replaced by fresh HBSS, and tissue fragments were subjected to vigorous mechanical dissociation. The isolated epithelial cells were passed through a 100-μm cell strainer (352360; Falcon) to remove larger fragments, washed in PBS, and the cells were either frozen for protein isolation or resuspended in Maxwell RNA homogenization solution (AS1340; Promega) for RNA purification and stored at -80°C until further processing.

### RNA Isolation, Complementary DNA Synthesis, and Quantitative Real-Time PCR

RNA was extracted using the Maxwell RSC simplyRNA Tissue Kit (AS1340) according to the manufacturer’s protocol and transcribed into complementary DNA using the High-Capacity Complementary DNA Reverse Transcription Kit (4368813; Thermo Fisher Scientific). Quantitative real-time PCR was performed on a Quant Studio 6 flex Thermocycler system (Thermo Fisher Scientific) with triplicate reactions using predesigned TaqMan assays (Thermo Fisher Scientific). Assay numbers of TaqMan probes used in this study are listed in Table 3, primers used for genotyping in Table 4, and kits used for RNA isolation and reverse transcription in Table 5. *Villin* and *Gapdh* were used for normalization and the expression levels were calculated using the Δ_Ct_ method and presented as –(ΔΔ_C__t_) values in the final figures.

**Table 3:** TaqMan Assays

Taq**M**an assays	Source	Identifier
Mouse ALPI real-time PCR gene expression assay FAM-MGB	Thermo Fisher Scientific	4331182/Mm01285814_g1
Mouse ASCL2 real-time PCR gene expression assay FAM-MGB	Thermo Fisher Scientific	4331182/Mm01268891_g1
Mouse ATOH1 real-time PCR gene expression assay FAM-MGB	Thermo Fisher Scientific	4331182/Mm00476035_s1
Mouse AXIN2 real-time PCR gene expression assay FAM-MGB	Thermo Fisher Scientific	4331182/Mm00443610_m1
Mouse CDX1 real-time PCR gene expression assay FAM-MGB	Thermo Fisher Scientific	4331182/Mm00438172_m1
Mouse chromogranin A real-time PCR gene expression assay FAM-MGB	Thermo Fisher Scientific	4331182/Mm00514341_m1
Mouse cyclin D1 real-time PCR gene expression assay FAM-MGB	Thermo Fisher Scientific	4331182/Mm00432359_m1
Mouse DLL1 real-time PCR gene expression assay FAM-MGB	Thermo Fisher Scientific	4331182/Mm01279269_m1
Mouse GAPD (GAPDH) endogenous control (VIC/MGB probe, primer limited)	Thermo Fisher Scientific	4352339E
Mouse HES1 real-time PCR gene expression assay FAM-MGB	Thermo Fisher Scientific	4331182/Mm01342805_m1
Mouse HSP90B1/GP96 exon 5–6 real-time PCR gene expression assay FAM-MGB	Thermo Fisher Scientific	4351372/Mm01253173_m1
Mouse LGR5 real-time PCR gene expression assay FAM-MGB	Thermo Fisher Scientific	4331182/Mm00438890_m1
Mouse LYZ1 real-time PCR gene expression assay FAM-MGB	Thermo Fisher Scientific	4331182/Mm00657323_m1
Mouse MKi67 real-time PCR gene expression assay FAM-MGB	Thermo Fisher Scientific	4331182/Mm01278617_m1
Mouse MUC2 real-time PCR gene expression assay FAM-MGB	Thermo Fisher Scientific	4331182/Mm01276696_m1
Mouse MYC real-time PCR gene expression assay, FAM MGB	Thermo Fisher Scientific	4331182/Mm00487804_m1
Mouse OLFM4 real-time PCR gene expression assay FAM-MGB	Thermo Fisher Scientific	4331182/Mm01320260_m1
Mouse REG4 real-time PCR gene expression assay FAM-MGB	Thermo Fisher Scientific	4331182/Mm00471115_m1

HSP90B1, heat shock protein 90 B1; MUC2,mucin 2; REG4, Regenerating Family Member 4.

**Table 4:** Primers

Primers	Source	Identifier	Concentration
Hsp90b1_5'arm	Microsynth	5'-GCCTGAAAGTCACACTCAACTTCC-3'	10 μm
Hsp90b1_3'arm	Microsynth	5'-CTACCCTACAGTCTATGTTATGGC-3'	10 μm
Hsp90b1_Tm1c_f	Microsynth	5'-AAGGCGCATAACGATACCAC-3'	10 μm
Hsp90b1_Tm1c_r	Microsynth	5'-CCGCCTACTGCGACTATAGAGA-3'	10 μm
VilCreERT2_1	Microsynth	5'-CAAGCCTGGCTCGACGGCC-3'	10 μm
VilCreERT2_2	Microsynth	5'-CGCGAACATCTTCAGGTTCT-3'	10 μm

Hsp, heat shock protein.

**Table 5:** Critical Commercial Assays

Critical commercial assays	Source	Identifier
CellTiter-Glo 3D cell viability assay	Promega	G9681
High-capacity cDNA reverse-transcription kit	Thermo Fisher Scientific	4368813
Maxwell RSC simplyRNA tissue kit	Promega	AS1340

### Western Blot Analysis

Proteins were extracted using M-PER lysis buffer (for IECs) or RIPA buffer (50 mmol/L Tris pH 7.5, 150 mmol/L NaCl, 1% NP-40, 0.5% sodium deoxycholate, 0.1% sodium dodecyl sulfate for organoid cultures) supplemented with a protease inhibitor cocktail (11836153001; Roche). After incubation for 1 hour on ice, the cell suspension was lysed by sonification for 3 times 10-seconds per sample and lysates were centrifuged at maximum speed for 10 minutes at 4° C to remove debris. Equal amounts of protein from each sample were separated by standard sodium dodecyl sulfate–polyacrylamide gel electrophoresis procedures and transferred onto nitrocellulose membranes (GE Healthcare). Membranes were blocked with 5% blocking solution (BSA or milk powder in washing buffer [Tris-buffered saline containing 1% Tween-20]) for 1 hour, followed by incubation with primary antibodies (diluted 1:1000 in blocking solution) overnight at 4°C. The primary and secondary antibodies used for Western blot are listed in Table 2. β-actin was used as loading control. Membranes were washed 3 times in Tris-buffered saline containing 1% Tween-20, and incubated for 1 hour at room temperature with horseradish peroxidase–labeled secondary antibodies (1:1000 in blocking solution), washed with Tris-buffered saline containing 1% Tween-20, and immunoreactive proteins were visualized using WesternBright ECL or SIRIUS horseradish peroxidase substrate (K-12045-D50, K-12043-D50; Advansta) and a Fusion Solo S Imager (Vilber). Images were analyzed using the Gel Analysis method in ImageJ software (National Institutes of Health).

### Organoid Preparation for Immunofluorescence Staining

For whole-mount organoid imaging, enteroids were grown on Lab-TekTM 8-chamber slides (154534; Thermo Fisher Scientific) and fixed with 4% paraformaldehyde, rinsed with immunofluorescence buffer (PBS pH 7.2, 0.2% Triton X-100 (Sigma-Aldrich), 0.05% Tween 20), permeabilized with 0.5% Triton X-100 in PBS, blocked in immunofluorescence buffer plus 10% normal goat serum, 1% BSA, and incubated with primary antibodies at 4°C overnight. Secondary antibodies were added for 2 hours together with 20 μg/mL 4′,6-diamidino-2-phenylindole (D1306; Invitrogen) and slides were mounted with Dako Fluorescent Mounting Medium (S3023). Table 2 contains details about the antibodies used.

### In Vitro Culture of Murine Intestinal Crypts

Duodenum, jejunum, or ileum sections were obtained from 8- to 17-week-old mice. The intestines were flushed with ice-cold PBS to remove debris, opened longitudinally, rinsed again with PBS, and cut into small fragments. The slices were washed extensively with PBS and incubated with Gentle Cell Dissociation Reagent (07174; Stemcell Technologies) for 20 minutes at room temperature on a roller shaker. Gentle Cell Dissociation Reagent was decanted, tissue pieces were agitated vigorously with ice-cold PBS + 0.1% BSA and the crypt-containing medium was passed through a 70-μm cell strainer (352350; Falcon) to remove villus fragments. The process was repeated 6 times. Crypt-containing fractions were centrifuged at 290 × *g* for 5 minutes at 4°C, washed with 0.1% BSA, and resuspended in cold Dulbecco’s modified Eagle medium/F12 (11330057; Thermo Fisher Scientific). The crypt-enriched pellets were resuspended in ice-cold Matrigel (FAL356231; Corning), 50-uL Matrigel drops were seeded into a prewarmed 24-well plate (3526; Corning), and overlaid with IntestiCult Organoid Growth Medium (06005; Stemcell Technologies) supplemented with 50 μg/mL penicillin-streptomycin (15140122; Thermo Fisher Scientific), and for the first 2–3 days additionally with 10 μmol/L Y-27632 (72302; Stemcell Technologies). The embedded crypts were cultured at 37°C and 5% CO_2_, growth media was replaced every 2–4 days, and mature organoids were passaged weekly by a combination of chemical and mechanical disruption at a splitting ratio of 1:2–3. For in vitro induction of Cre recombinase activity, organoids were cultured for 2–4 days before treatment with 1 μmol/L 4-OHT (T176; Sigma); dimethyl sulfoxide was used as control. 4-OHT–containing medium was removed after 2 days and replaced by fresh complete growth media. In designated experiments, culture media was supplemented additionally with 5 μmol/L CHIR99021 (CHIR) (4423/10; Tocris), 10 μmol/L DAPT (2634; Tocris), 10 μmol/L XAV939 (XAV) (ab120897; Abcam), or 1 mmol/L VPA (2815; Tocris). At the indicated times, organoids were collected and processed as described earlier. Biological replicates were defined as organoid lines generated from different mice with the same genotype or a repetition of an experiment at least 1 passage apart. Separate wells were considered as technical replicates. For certain experimental readouts, 2–3 wells (ie, real-time PCR) were pooled for analysis. Brightfield live-imaging of organoids was performed using a Nikon Eclipse Ts2 inverted microscope equipped with a Nikon DS-Fi3 camera and images were edited with ImageJ software (National Institutes of Health).

### Isolation of Intestinal Stem Cells

For intestinal stem cell isolation, crypts were isolated as described earlier. Crypts then were resuspended in HBSS containing 3 mmol/L EDTA and dissociated for 15 minutes on a shaker at 300 rpm and 37°C. The cells then were passed through a 70-μm cell strainer, washed in PBS, and CD44^+^CD24^lo^CD166^+^ GRP78^lo/-^ intestinal stem cells were sorted as described by Wang et al^48^ using a BD Aria III sorter. After sorting, the cells were washed with 0.1% BSA-containing Dulbecco’s modified Eagle medium/F12. The cells were resuspended in ice-cold Matrigel, 50-uL Matrigel drops were seeded into a prewarmed 24-well plate, and overlaid with IntestiCult Organoid Growth Medium supplemented with 50 μg/mL penicillin-streptomycin and 1 μmol/L 4-hydroxytamoxifen. After 24, 48, 72, and 96 hours, the cells were harvested for RNA and protein isolation. For viability assessment, the cells were seeded in 96-well plates and viability was assessed after 24, 48, 72, and 96 hours, as described later.

### Viability Assay

For assessing organoid growth and viability, organoids were seeded in 96-well plates. After 7 days in culture, the CellTiter-Glo 3D Cell Viability Assay (G9681; Promega, see Table 5) was used to determine viability according to the manufacturer’s direction and luminescence was detected using a BioTek Synergy H1 microplate reader. Assays were performed with 5 replicates for 3 independent experiments and wells containing Matrigel domes and media but no organoids served as blanks.

### Single Nucleotide Polymorphism (SNP) Analysis

Data on genetic variants were obtained from the Genome Aggregation Database (https://gnomad.broadinstitute.org).

### Statistical Analysis

Statistical analysis was conducted using GraphPad Prism 8 software (GraphPad, San Diego, CA). Statistical comparisons between groups were performed using 1-way or 2-way analysis of variance with appropriate statistical tests to correct for multiple comparisons as specified in the figure legends. Specific numbers of biological replicates are noted in each figure legend. Data are shown as means ± SD. *P* ≤ .05 was considered statistically significant with a 95% CI.

All authors were able to access the study data and reviewed and approved the final manuscript.
